# Supplementary material for: Upregulation of β-catenin due to loss of miR-139 contributes to motor neuron death in amyotrophic lateral sclerosis
Source: Stem Cell Reports. 2022 Jun 23;17(7):1650–65. doi: 10.1016/j.stemcr.2022.05.019 (PMC9287677; doi:10.1016/j.stemcr.2022.05.019)
Supplement: Document S1. Figures S1–S5, Tables S1–S4, and supplemental experimental procedures [file mmc1.pdf]

**Stem Cell Reports, Volume *17***

## Supplemental Information

# Upregulation of β-catenin due to loss of miR-139 contributes to motor neuron death in amyotrophic lateral sclerosis

**Sophie Hawkins, Seema C. Namboori, Ammarah Tariq, Catherine Blaker, Christine Flaxman, Nidhi S. Dey, Peter Henley, Andrew Randall, Alessandro Rosa, Lawrence W. Stanton, and Akshay Bhinge**

Figure S1

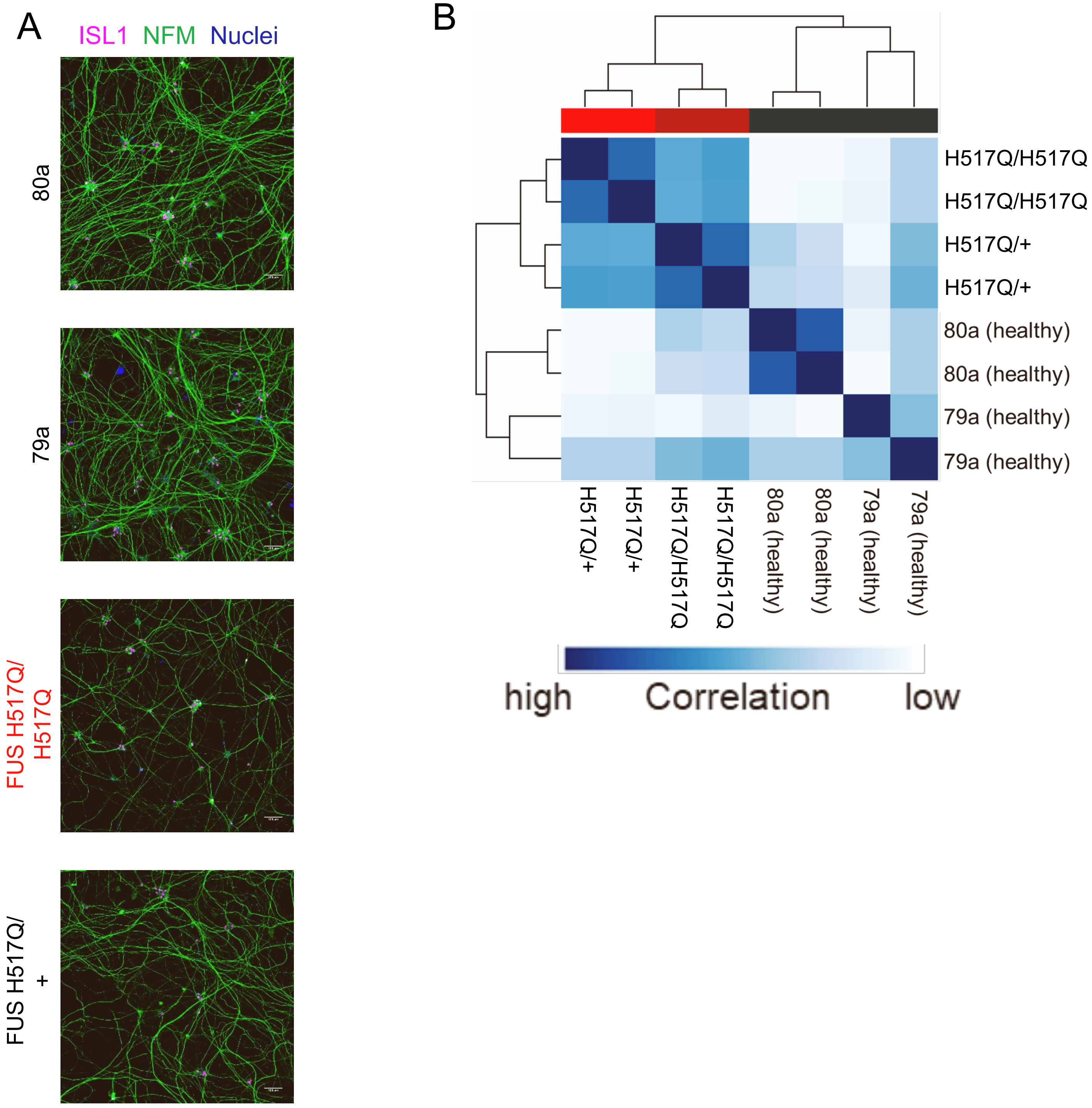


**Figure S1. Related to figures 1 and 2.**
**A)** Representative images of healthy (80a and 79a) MNs and FUS ALS (FUS H517Q/H517Q and FUS H517Q/+) at day 45. Scale bar indicates 100um. **B).** Hierarchical cluster analysis of 844 miRNAs in FUS H517Q/H517Q, FUS H517Q/+ and healthy MNs. N = 2 independent differentiations.

Figure S2

A

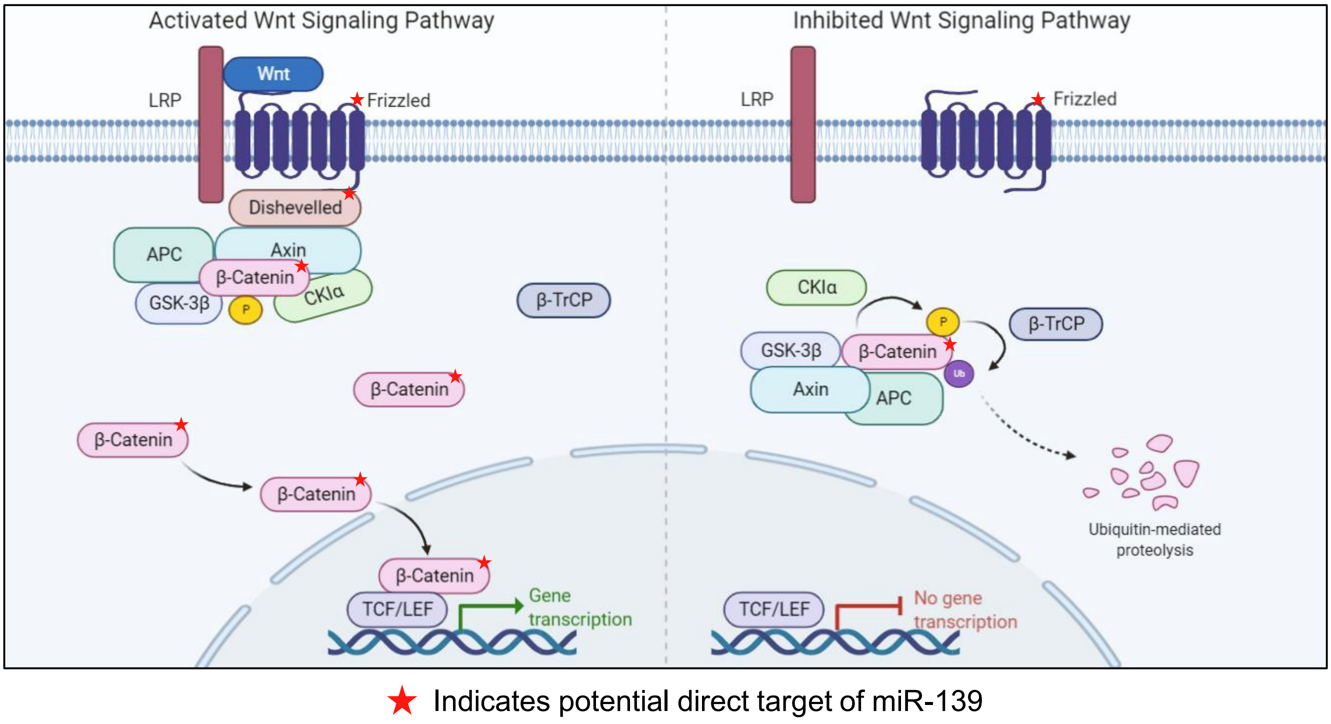


B

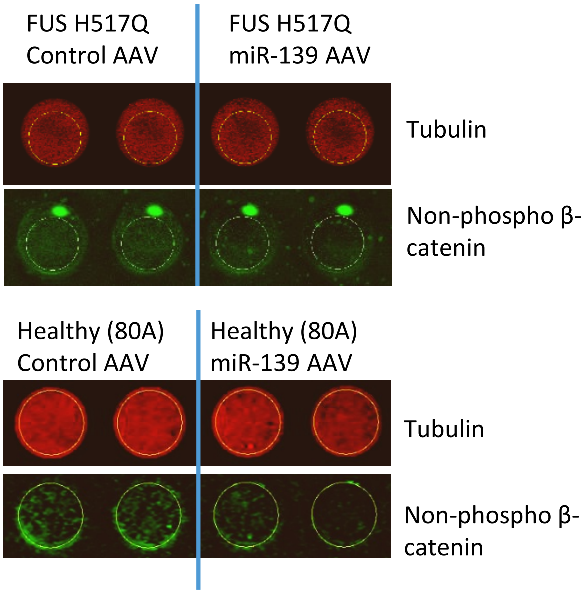


C

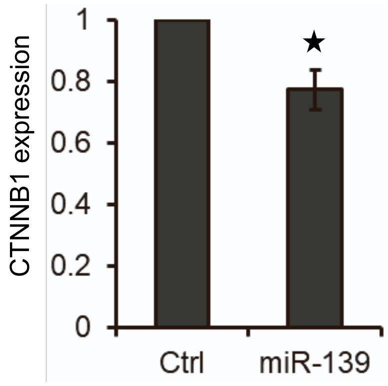


**Figure S2. Related to figure 3.**
**A)** Schematic of activated and inhibited canonical WNT signalling (left and right panels respectively). Canonical WNT signalling is activated by the binding of WNT ligands to frizzled and LRP5/6 co-receptors on the cell surface. This leads to the recruitment and sequestration of the β-catenin destruction complex to the membrane, preventing further phosphorylation and ubiquitin-mediated destruction. β-catenin accumulates and translocates to the nucleus where it binds to TCF and promotes transcription of downstream target genes. Image created with Biorender. Stars indicate the top three potential direct targets of miR-139 displayed in Fig.3E. **B)** Representative images of in-cell western blots in figure 3F. The circles indicate the area used for quantification. **C)** RT-qPCR analysis of CTNNB1 / β-catenin transcript in Day 34 FUS H517Q/H517Q MNs in response to miR-139 overexpression. N = 3 independent differentiations. Error bars indicate SEM. * indicates p-value < 0.05.

Figure S3

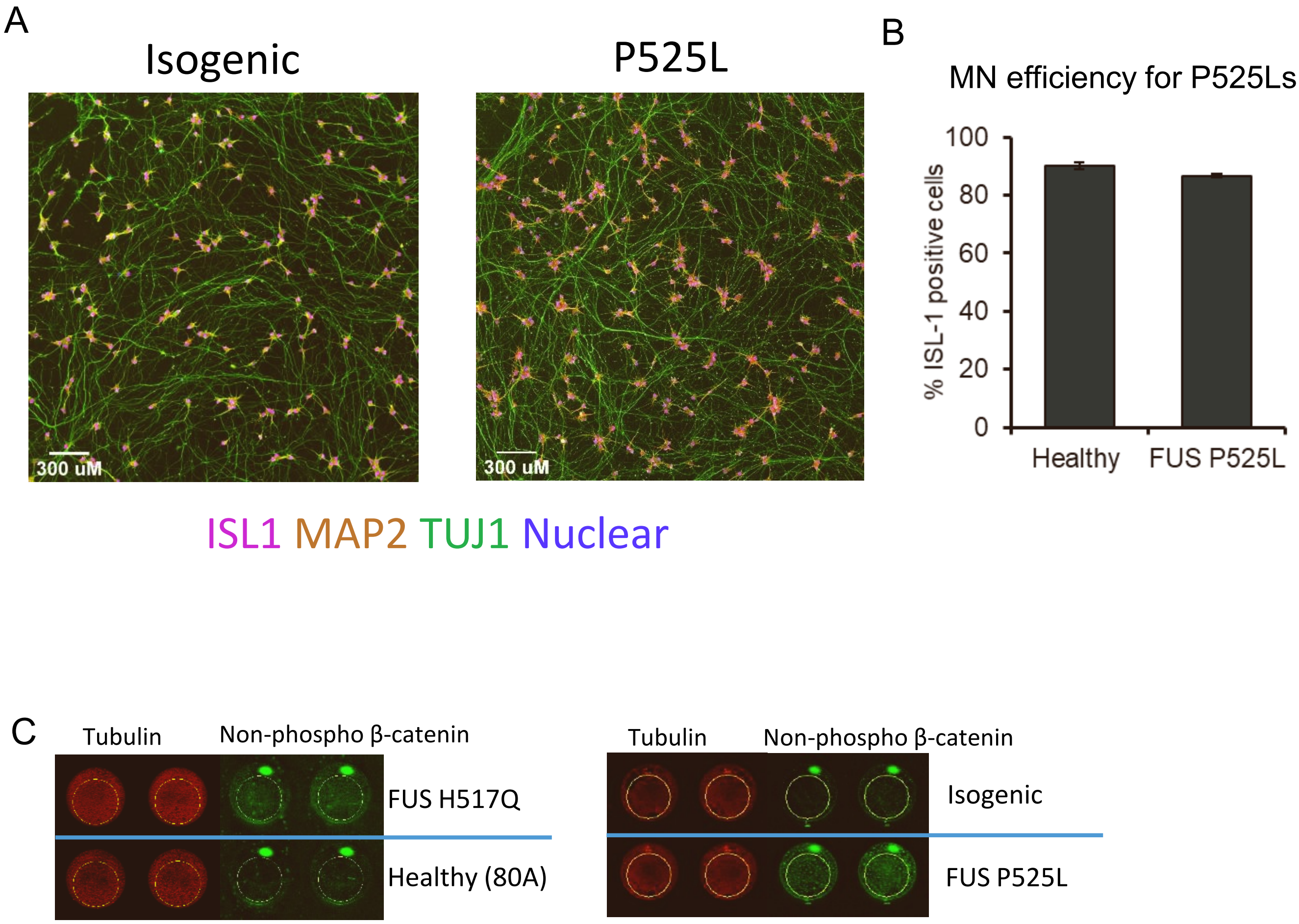


**Figure S3: Related to figure 4**

**A)** Representative images: Isogenic and P525L MNs immunostained with ISL1, MAP2 and TUJ1. Scale bar indicates 300uM. **B)** MN differentiation efficiency, determined by ISL-1+ nuclei. N = 3 independent differentiations. **C)** Representative images for in-cell western blot. Left panel = FUS H517Q/H517Q vs healthy MNs (80A). Right panel = FUS P525L vs isogenic control MNs. Circles indicate area used for quantification.

Figure S4

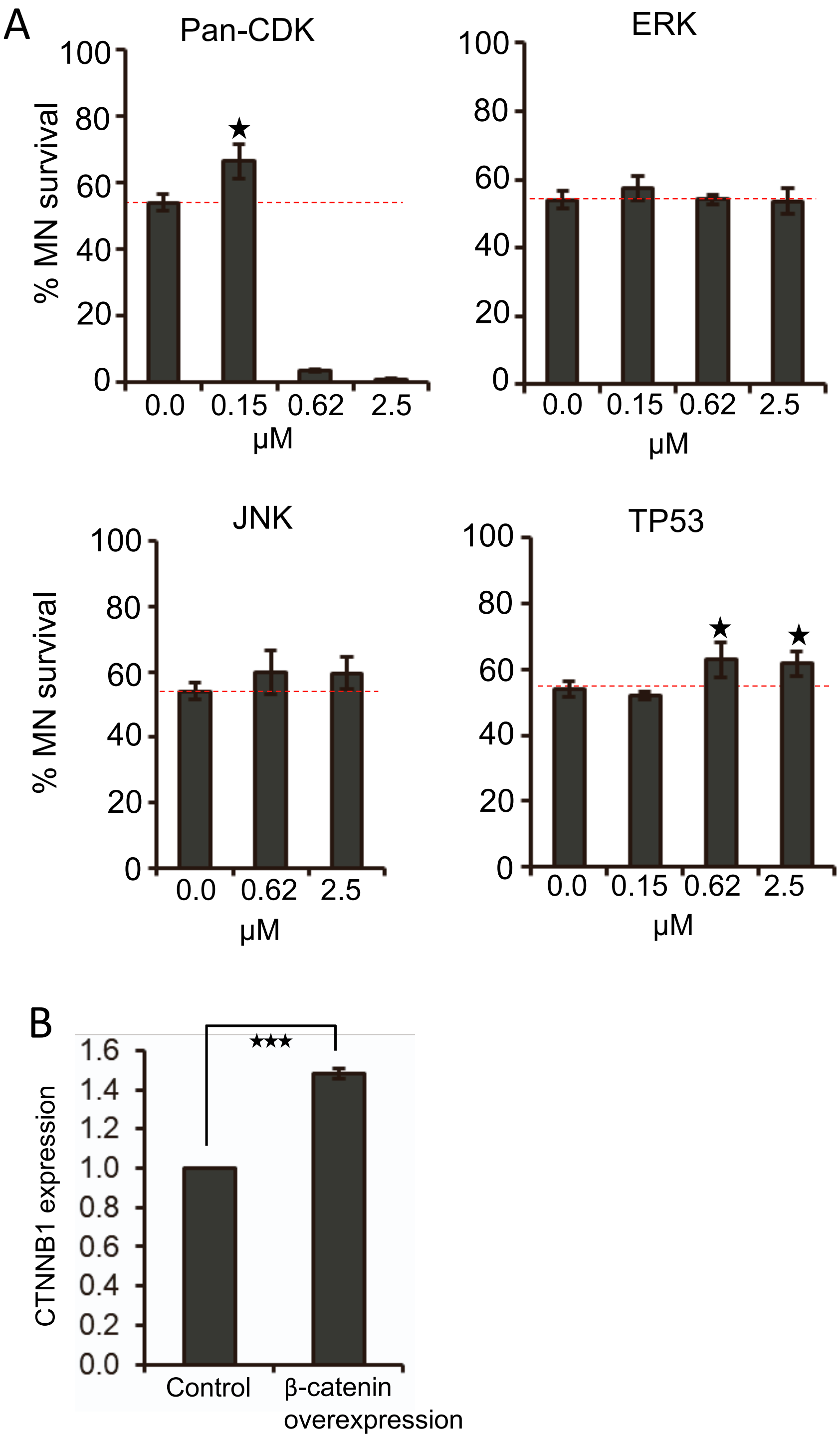


**Figure S4: Related to figure 4, role of WNT in FUS ALS MN degeneration**
**A)** Quantification of ISL1 positive FUS H517Q/H517Q MN at day 45 relative to day 30 after treatment with small molecule inhibitors of the indicated pathways. Horizontal dotted line indicates MN loss in the DMSO only control. N = 3 independent differentiations. **B)** Quantitative RT-qPCR indicating upregulation of CTNNB1 transcript 48 hours post-transduction in healthy (80A) MN progenitors. N = 3.
N indicates the number of independent differentiations. * indicates p-value < 0.05, ** indicates p-value < 0.01. P-values were estimated by Student's two-tailed t-test.

# Figure S5

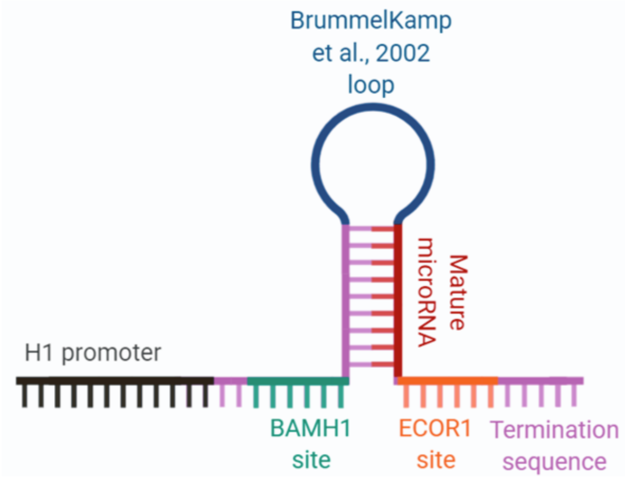


**Figure S5.** Schematic depicting shRNA design for miR-139-5p overexpression

| Antibody | Species | Brand / identifier | Dilution |
|---|---|---|---|
| ISL-1 | Ms / Rb | Abcam ab86501 / ab108517 | 1:500 |
| NF-H | Rb | Sigma N4142 | 1:1000 |
| MAP2 | Ms / Rb | Abcam ab11267 / ab32454 | 1:1000 |
| TUJ1 | Ck | Abcam ab107216 | 1:1000 |
| Total β-catenin | Rb | Abcam ab16051 | 1:500<br>1:4000 |
| Non-phospo β-catenin | Rb | CST 8814 | 1:500 |
| Tubulin | Ms | Abcam ab7291 | 1:1000 |

**Table S1.** Primary antibodies used for immunofluorescence and western blot

| Target | Forward 5’ to 3’ | Reverse 5’ to 3’ |
|---|---|---|
| AXIN2 | GTGAGGTCCACGGAAACTGT | TGGCTGGTGCAAAGACATAG |
| DKK1 | CCTTGGATGGGTATTCCAGA | CCTGAGGCACAGTCTGATGA |
| CCND3 | TTGCACATGATTTCCTGGCC | ACTTCAGTGCCAGTGATCCC |
| WNT3 | CGCACGACTATCCTGGAC | GAGGCGCTGTCATACTTGTC |
| WNT4 | CTAGCCCCGACTTCTGTGAG | TTGGACGTCTTGTTGCATGT |
| WNT5B | CGGGAGCGAGAGAAGAACT | CGTCTGCCATCTTATACACAGC |
| WNT10B | GATACCCACAACCGCAATTC | GGGTCTCGCTCACAGAAGTC |
| WNT11 | GGCCAAGTTTTCCGATGCTC | CACCCCATGGCACTTACACT |
| CTNNB1 | CACAAGCAGAGTGCTGAAGGTG | GATTCCTGAGAGTCCAAAGACAG |
| HPRT1 | CATTATGCTGAGGATTTGGAAAGG | CTTGAGCACACAGAGGGCTACA |
| RPL13 | CCTGGAGGAGAAGAGGAAAGAGA | TTGAGGACCTCTGTGTATTTGTCAA |

**Table S2.** Primer sequences for mRNA qPCR

| Target | 5’ to 3’ |
|---|---|
| Universal R | GCATAGACCTGAATGGCGGTA |
| hsa-miR-139-5p | CAGTGCACGTGTCTCCAGTAAAA |
| SNORD44 | GCAAATGCTGACTGAACATGAA |
| SNORD48 | CTCTGAGTGTGTCGCTGATGC |
| RNU19 | GGAATGACTCCTGTGGAGTTGA |

**Table S3.** Primer sequences for microRNA qPCR

| Chemical | Source | Pathway / Target |
|---|---|---|
| Pifithrin-α hydrobromide | Tocris | TP53 |
| SB203580 | Tocris | p38/MAPK |
| FR180204 | SCBT | ERK 1,2 |
| SP600125 | SCBT | JNK 1,2,3 |
| XAV 939 | Tocris | WNT |
| AT7519 | Selleckchem | CDK1,2,3,4,6,9 |

**Table S4.** Small molecule inhibitors

**Supplemental experimental procedures**

**Human iPSC culture**

ALS patient-derived FUS H517Q/H517Q iPSC (ND35663), and healthy iPSCs (GM23280A, GM23279A) were obtained from the Coriell Institute for Medical Research. FUS H517Q/H517Q iPSCs were genome edited using CRISPR to generate the FUS H517Q/+ heterozygous cell line. All iPSC lines were maintained as colonies on human ES qualified Matrigel (Corning) in StemFlex (StemCell Technologies). Colonies were routinely passaged in a 1:6 split using EDTA. Mycoplasma testing was conducted regularly to rule out contamination of cultures.

**Differentiation of iPSC into spinal motor neurons**

iPSC were plated as colonies onto Matrigel and differentiated by treatment with neuronal differentiation media (DMEM/F12:Neurobasal in a 1:1 ratio, HEPES 10mM, N2 supplement 1%, B27 supplement 1%, L-glutamine 1%, ascorbic acid 5uM, insulin 20ug/ml) supplemented with SB431542 (40uM), CHIR9921 (3uM) and LDN8312 (0.2uM) from day 0 till day 4. Cells were caudalized by treatment with 0.1uM retinoic acid starting at day 2 and ventralized with 1uM purmorphamine starting at day 4 and continued till day 8. At day 8, motor neuron progenitors were re-plated onto poly-D-lysine/laminin coated wells and differentiation was induced by treating the cells with N2B27 media supplemented with retinoic acid, purmorphamine and and DAPT 10uM. DAPT treatment was stopped at day 13 and media was changed to N2B27 supplemented with BDNF 10ng/ml, GDNF 10ng/ml. Next day (day 14) neuronal cultures were pulsed with mitomycin at a dose of 10ug/ml for 1 hour to prevent further proliferation of any undifferentiated progenitors. Neuronal cultures were maintained by changing half the media every 2-3 days.

**Intracellular $Ca^{2+}$ imaging**

Day 30 MNs were plated onto poly-D-lysine and laminin coated cover slips that had been sterilized by autoclaving. Cells were loaded with the $Ca^{2+}$ sensitive dye Fura2-AM at a final concentration of 4μg/mL for 60 min at 37°C in a HEPES-buffered saline solution (HBSS) consisting of (140mM NaCl, 3mM KCl, 2mM CaCl2, 1mM MgCl2, 2.5mM Glucose, 10mM HEPES-NaOH, pH 7.35).

Coverslips were placed in a chamber on the stage of an inverted microscope (Nikon, 11 Eclipse TE2000-S). The cells were imaged (20x magnification) whilst constantly being perfused with HBSS (~1.4 mL/min). Every 5 seconds, Fura-2 was excited in succession at wavelengths of 340 and 380 nm using a high speed wavelength switcher (Lambda DG-4); the emitted fluorescence images at 510 nM were collected using a CCD camera (Hamamatsu Photonics). After a baseline period, neurons were treated for 2 min with 50μM glutamate dissolved the perfusing HBSS solution. The timeseries of the emission ratio from 340 and 380 excitation, which is proportion to intracellular $Ca^{2+}$, was calculated as described previously (Telezhkin *et al*, 2016). Data was collected in Volocity software (PerkinElmer) and plotted in Origin Pro.

**Immunostaining**

Cells were fixed with 4% paraformaldehyde, permeabilized with ice-cold methanol for 5 minutes and washed with PBS containing 10% serum for 1 hour at room temperature. Cells were incubated with primary antibodies (Table S1) diluted into PBS containing 1% BSA and incubated overnight at 4°C. Next day, cells were washed and incubated with Alexa-fluor conjugated secondary antibodies (Molecular probes) for 45 minutes at room temperature and nuclei were stained with Hoechst 33542 (Molecular probes). Images were obtained in an automated fashion on the ImageXpress Pico (Molecular devices). Nuclear identification and ISL1 nuclear signal was quantified in an automated fashion using ImageXpress software. Threshold intensities were maintained the same across wells.

**MN survival assay**

Motor neuron progenitors were plated in 96-well optically clear black tissue culture plates (Greiner). Day 30 cultures were fixed and stained for ISL1 to assess MN counts. A separate plate cultured under the same condition was allowed to proceed till day 45, when it was fixed and stained for ISL1. Nuclei were stained using Hoechst 33342. MN counts at day 45 were compared with day 30 to assess MN survival. We performed three independent differentiations with two technical wells used per replicate. Data from the technical wells was pooled to generate counts for each replicate. Apoptotic cells at day 37 were detected using the CellEvent Caspase 3/7 green detection reagent (Thermofisher) according to manufacturer's instructions. Drug treatments (Table S4) were initiated at day 30 and MN survival performed as described above.

**Quantitative RT-PCR**

Total RNA was extracted with the miRNeasy kit (Qiagen) or Monarch Total RNA miniprep (NEB) and reverse transcribed using random hexamers and the High Capacity reverse transcription system from Applied Biosystems. Quantitative PCR was performed using the SYBR GREEN PCR Master Mix from Applied Biosystems. The target gene mRNA expression was normalized to the expression of two housekeeping genes (HPRT1 and RPL13), and relative mRNA fold changes were calculated by the ΔΔCt method. Primer sequences are included in Table S2.

**Western blot analysis**

Cell lysates were prepared in RIPA buffer, separated on 12% SDS-PAGE gels and proteins were transferred onto PVDF membranes. Membranes were blocked with 5% milk in TBST (25 mM Tris pH 8.0, 150 mM NaCl, 0.05% Tween-20) and probed with corresponding primary antibodies against specific proteins (Table S1). Dylight 680 and 800 secondary antibodies

(Cell signalling technology) were used to detect primary antibodies and proteins were visualized by using fluorescence (Licor).

**In-cell western assay**

Cells were fixed with 4% paraformaldehyde, permeabilized with ice-cold methanol for 5 minutes and washed with PBS containing 10% serum for 1 hour at room temperature. Cells were incubated with primary antibodies (Table S1) diluted into PBS containing 1% BSA and incubated overnight at 4°C. Next day, cells were washed and incubated with Dylight 680 and 800 secondary antibodies (Cell signalling technology) for 1 hr at room temperature. Fluorescence was captured with the Odyssey CLx Imaging System (LI-COR) according to the manufacturer's instructions.

**MicroRNA RT-qPCR**

Total RNA was reverse transcribed using the qScript microRNA cDNA Synthesis Kit (QuantaBio). Quantitative PCR was performed with SYBR GREEN PCR Master Mix from Applied Biosystems and fold changes were calculated by the ΔΔCt method. Primer sequences are included in Table S3.

**Caspase 3/7 Assay**

Caspase was measured using the Promega Caspase-Glo® 3/7 Assay System kit (G8090). Caspase luminescence is presented relative to cell titer, as measured by Promega CellTiter-Glo® 2.0 Cell Viability Assay.

**FUS Knockdown**

300,000 SH-SY5Y or MN progenitor cells were nucleofected with 30 pmol silencer select siRNA (Thermo Fisher) against FUS or appropriate scrambled control using the P3 Primary nucleofector kit (Lonza).

**RNA sequencing and analysis**

RNA was extracted from day 30 neuronal cultures as described above. MicroRNA sequencing libraries were generated from 500ng of RNA using the NEBnext Small RNA kit (NEB) according to manufacturer's instructions and sequenced on an Illumina MiSeq instrument. Raw reads were trimmed to remove adapter sequences, and PCR duplicates collapsed using the fastx toolkit (http://hannonlab.cshl.edu/fastx_toolkit/index.html). Processed reads were mapped to the microRNA hairpin sequences obtained from miRbase using bowtie (Langmead *et al*, 2009) and custom scripts were used to generate counts per microRNA.

Total RNA extracted as described above was used to generate Illumina libraries for whole transcriptome analysis using the NEBNext Ultra II kit (NEB). RNA-sequencing reads were trimmed using cutadapt (Martin, 2011) and mapped to the human genome hg19 using bwa (Li & Durbin, 2009). Only reads mapping to unique locations were retained for further analysis.

For the miRNA OE analysis, total RNA was extracted at day 35. RNA was processed using the Quant-seq kit (Lexogen) to generate 3'end libraries compatible with Illumina. Reads were trimmed using cutadapt and mapped to the hg19 using STAR (Dobin *et al*, 2013).

The DESeq2 R package (Love *et al*, 2014) and custom R scripts were used to analyze the read counts and generate a list of differentially expressed genes sorted by the statistical score. Gene set enrichment analysis was performed using the GSEA software (http://software.broadinstitute.org/gsea/index).

**Pri-miRNA quantification**

We sought to determine pri-miRNA transcript levels from RNA-seq data. Over 60% of all mapped miRNAs to date are intragenic, residing within introns of host genes (Hinske *et al*, 2017). Since most intragenic miRNAs display correlated expression with their host genes (Liu

*et al*, 2018), we decided to use the host gene expression as a proxy for pri-miRNA expression levels. Standard RNA-seq experiments generate reads that can be classified as intronic or exonic based on whether they map to introns or exons respectively. Since introns are absent in cytosolic mRNAs, RNA-Seq reads mapping to introns can be used to represent levels of unspliced transcripts present in the nucleus. Changes in intronic read counts can be used to measure changes in transcriptional output for a given gene across experimental conditions (Gaidatzis *et al*, 2015). Hence, we measured intronic reads mapping to host genes as estimates of pri-miRNA transcription.

Intronic miRNAs were identified from the miRIAD intragenic microRNA database (Hinske *et al*, 2014). MiRNAs and host genes were shortlisted to those that were expressed in our MN datasets. Custom R scripts were used to quantify reads mapping to intronic regions of host genes from whole transcriptome libraries. Reads were normalised using scale factors generated with DESeq2.

## References


Dobin A, Davis CA, Schlesinger F, Drenkow J, Zaleski C, Jha S, Batut P, Chaisson M & Gingeras TR (2013) STAR: ultrafast universal RNA-seq aligner. *Bioinformatics* 29: 15–21

Gaidatzis D, Burger L, Florescu M & Stadler MB (2015) Analysis of intronic and exonic reads in RNA-seq data characterizes transcriptional and post-transcriptional regulation. *Nat Biotechnol* 33: 722–729

Hinske LC, Dos Santos FRC, Ohara DT, Ohno-Machado L, Kreth S & Galante PAF (2017) MiRIAD update: using alternative polyadenylation, protein interaction network analysis and additional species to enhance exploration of the role of intragenic miRNAs and their host genes. *Database* 2017

Hinske LC, França GS, Torres HAM, Ohara DT, Lopes-Ramos CM, Heyn J, Reis LFL, Ohno-Machado L, Kreth S & Galante PAF (2014) miRIAD-integrating microRNA inter- and intragenic data. *Database* 2014

Langmead B, Trapnell C, Pop M & Salzberg SL (2009) Ultrafast and memory-efficient alignment of short DNA sequences to the human genome. *Genome Biol* 10: R25

Li H & Durbin R (2009) Fast and accurate short read alignment with Burrows-Wheeler transform. *Bioinformatics* 25: 1754–1760

Liu B, Shyr Y, Cai J & Liu Q (2018) Interplay between miRNAs and host genes and their role

in cancer. *Brief Funct Genomics* 18: 255–266

Love MI, Huber W & Anders S (2014) Moderated estimation of fold change and dispersion for RNA-seq data with DESeq2. *Genome Biol* 15: 550

Martin M (2011) Cutadapt removes adapter sequences from high-throughput sequencing reads. *EMBnet.journal* 17: 10–12

Telezhkin V, Schnell C, Yarova P, Yung S, Cope E, Hughes A, Thompson BA, Sanders P, Geater C, Hancock JM, *et al* (2016) Forced cell cycle exit and modulation of GABAA, CREB, and GSK3β signaling promote functional maturation of induced pluripotent stem cell-derived neurons. *Am J Physiol Cell Physiol* 310: C520–41
