# Supplementary material for: Upregulation of β-catenin due to loss of miR-139 contributes to motor neuron death in amyotrophic lateral sclerosis
Source: Stem Cell Reports. 2022 Jun 23;17(7):1650–65. doi: 10.1016/j.stemcr.2022.05.019 (PMC9287677; doi:10.1016/j.stemcr.2022.05.019)
Supplement: Document S2. Article plus supplemental information [file mmc2.pdf]

Stem Cell Reports
Article
OPEN ACCESS

Upregulation of β-catenin due to loss of miR-139 contributes to motor neuron death in amyotrophic lateral sclerosis

Sophie Hawkins,[1,2] Seema C. Namboori,[1,2] Ammarah Tariq,[2,3] Catherine Blaker,[1] Christine Flaxman,[1] Nidhi S. Dey,[4] Peter Henley,[1] Andrew Randall,[1] Alessandro Rosa,[5,6] Lawrence W. Stanton,[7] and Akshay Bhinge[1,2,*]

[1]College of Medicine and Health, University of Exeter, Exeter EX1 2LU, UK
[2]Living Systems Institute, University of Exeter, Exeter EX4 4QD, UK
[3]Biosciences, University of Exeter, Exeter EX4 4QD, UK
[4]York Biomedical Research Institute, Hull York Medical School, University of York, York YO10 5DD, UK
[5]Department of Biology and Biotechnologies Charles Darwin, Sapienza University of Rome, P.le A. Moro 5, 00185 Rome, Italy
[6]Center for Life Nano- & Neuro-Science, Fondazione Istituto Italiano di Tecnologia (IIT), 00161 Rome, Italy
[7]Qatar Biomedical Research Institute, Hamad Bin Khalifa University, Doha, Qatar
*Correspondence: a.bhinge@exeter.ac.uk
https://doi.org/10.1016/j.stemcr.2022.05.019

SUMMARY

Amyotrophic lateral sclerosis (ALS) is a fatal neurodegenerative disease characterized by the loss of motor neurons (MNs). There are no effective treatments and patients usually die within 2–5 years of diagnosis. Emerging commonalities between familial and sporadic cases of this complex multifactorial disorder include disruption to RNA processing and cytoplasmic inclusion bodies containing TDP-43 and/or FUS protein aggregates. Both TDP-43 and FUS have been implicated in RNA processing functions, including microRNA biogenesis, transcription, and splicing. In this study, we explore the misexpression of microRNAs in an iPSC-based disease model of FUS ALS. We identify the downregulation of miR-139, an MN-enriched microRNA, in FUS and sporadic ALS MN. We discover that miR-139 downregulation leads to the activation of canonical WNT signaling and demonstrate that the WNT transcriptional mediator β-catenin is a major driver of MN degeneration in ALS. Our results highlight the importance of homeostatic RNA networks in ALS.

INTRODUCTION

Amyotrophic lateral sclerosis (ALS) is a fatal neurodegenerative disease, characterized by the loss of upper and lower motor neurons (MNs) that affects 1–2/100,000 individuals worldwide (Xu et al., 2020). From clinical diagnosis, the median survival rate is 2–5 years (Brown and Al-Chalabi, 2017), with death commonly due to respiratory distress. To date, there are only two approved treatments: riluzole and edaravone, which add a few months to life (Jaiswal, 2019). Developing effective treatments has proved difficult due to the inherent complexity of ALS and unclear disease mechanisms.

Most ALS cases are not familial (fALS) and are termed sporadic (sALS). Many genes linked to fALS, including *C9ORF72*, *TARDBP*, and *FUS*, are involved in RNA processing (Butti and Patten, 2018), with the disruption of RNA processing seen in both fALS and sALS (Tank et al., 2018). Molecular hallmarks of fALS and sALS include the presence of TDP-43 and/or FUS cytoplasmic inclusion bodies within MN (Ikenaka et al., 2020; Deng et al., 2010). TDP-43 and FUS primarily function in RNA processing events, such as transcription, alternative splicing, and microRNA (miRNA) biogenesis (Butti and Patten, 2018). Significantly, RNA processing defects, such as intron retention (Luisier et al., 2018) and mRNA instability (Tank et al., 2018), have been observed in FUS or TDP-43 fALS as well as sALS MN, indicating that ALS models harboring these mutations may provide insights into the molecular mechanisms of both fALS and sALS.

Patient-derived induced pluripotent stem cells (iPSCs) offer an excellent opportunity to create human *in vitro* models of disorders like ALS that are otherwise difficult to study due to diseased cell type inaccessibility. These models provide a powerful platform to study pathogenesis in the context of the genetic background of a patient, without the need to overexpress the mutant proteins (Hawrot et al., 2020).

In this study, we deployed iPSC-based disease modeling with transcriptomic and phenotypic analysis to investigate molecular drivers of FUS ALS MN degeneration. Our results indicate that the downregulation of the MN-specific miRNA miR-139 contributes to the activation of canonical WNT signaling that drives MN degeneration. Importantly, these molecular defects are also observed in sALS MN, highlighting the shared dysregulation of RNA metabolism in fALS and sALS.

RESULTS

Patient-derived FUS MN display disease-associated phenotypes

We used iPSCs carrying the homozygous H517Q mutation in the *FUS* gene (H517Q/H517Q) and CRISPR-Cas9-edited

 © 2022 The Author(s).
This is an open access article under the CC BY license (http://creativecommons.org/licenses/by/4.0/).

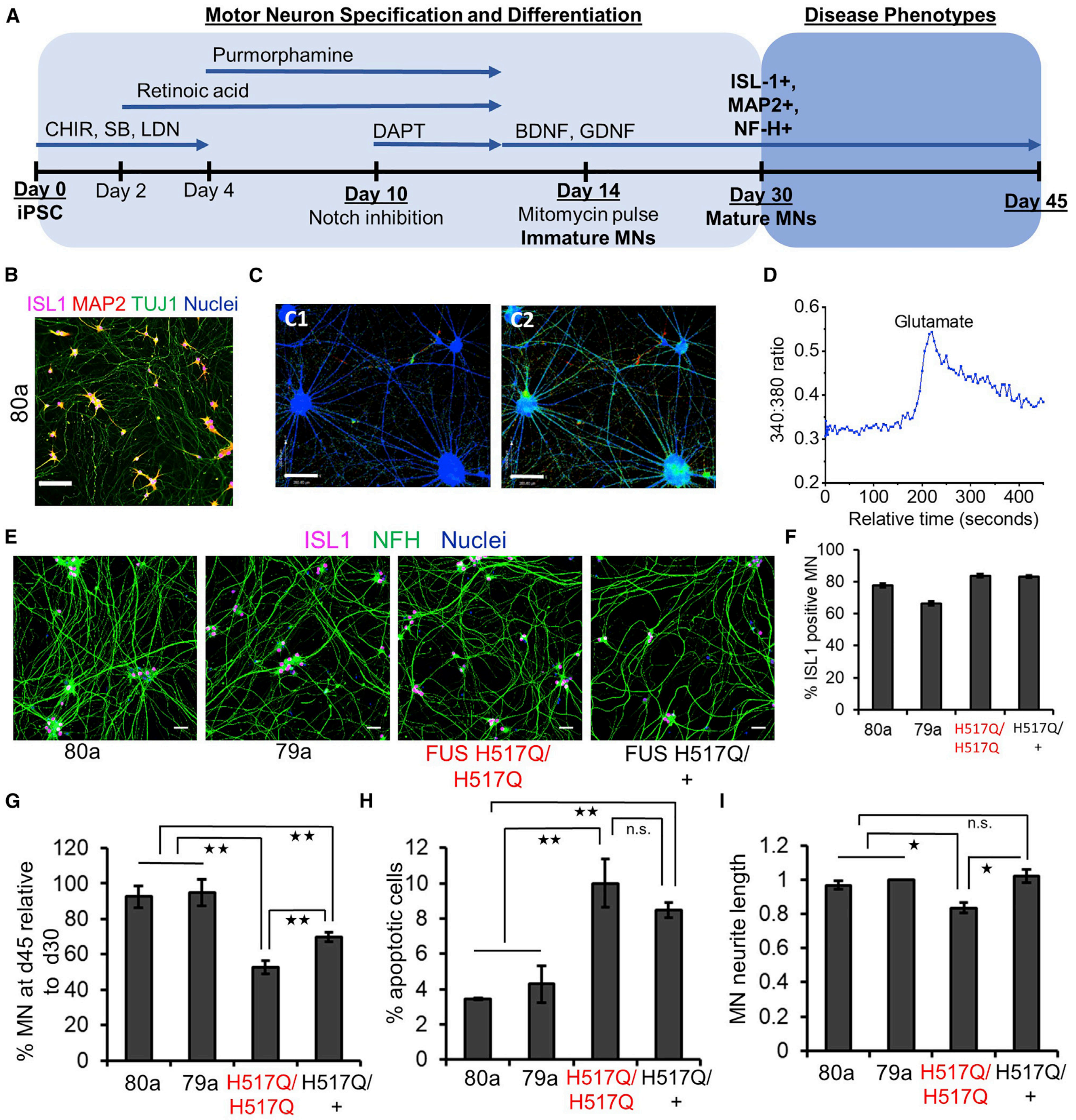

Figure 1. MN derived from FUS-ALS iPSC display disease-related phenotypes

80a, 79a indicate healthy iPSCs.

H517Q/H517Q, H517Q/+ indicate ALS FUS iPSCs with a homozygous mutation or heterozygous correction of the mutation, respectively.

(A) Schematic depicting differentiation to MN from human iPSCs. See supplemental experimental procedures for further details. CHIR: CHIR09921, SB: SB431542, LDN: LDN193189.

(B) Representative image: 80a MN immunostained with ISL1, MAP2, and TUJ1 at day 34. Scale bar indicates 300 μm.

(C) 80a day 34 MN captured during ratiometric Fura-2 Ca^{2+} imaging. C1: Baseline level before stimulation. C2: Elevated Ca^{2+} during stimulation with 50 μM glutamate. Scale bar indicates 200 μm.

(D) Time course of MN response to glutamate. X axis indicates relative time in seconds. Y axis indicates the whole field average (WFA) 340:380 emission ratio for Fura-2, which is proportional to intracellular Ca^{2+} concentration.

(legend continued on next page)

heterozygous iPSC (H517Q/+) derived from this line (Bhinge et al., 2017), as well as two healthy iPSC lines to evaluate disease-associated phenotypes in the FUS ALS MN. We deployed a robust protocol to differentiate all iPSCs into spinal MN (Figure 1A). Day 30 neurons expressed the mature MN markers ISL1, MAP2, NF-H, and TUJ1 (Figures 1B and 1E). In addition, our iPSC-derived MN demonstrated an increase in intracellular Ca^{2+} concentration in response to glutamate stimulation, indicating expression of glutamate receptors, a key feature of functionally mature MNs (Figures 1C and 1D). Across five independent differentiations, all four iPSC lines could be differentiated to $ISL1^{+}$ MN at an efficiency of ~80% (Figures 1E and 1F), validating the reproducibility of our protocol.

We assessed MN survival by counting $ISL1^{+}$ nuclei at days 30 and 45 for all of the genotypes. FUS MNs displayed >50% loss *in vitro*, while the healthy MNs displayed <15% loss (Figures 1G and S1A). FUS MN also displayed higher levels of apoptotic cells compared to healthy MN (Figure 1H). Heterozygous correction of the mutation rescued the survival deficit in FUS MN (Figures 1G, 1H, and S1A), indicating that the neuronal loss was due to the FUS H517Q mutation. However, the survival deficits in the H517Q/+ MN were significantly higher than in the healthy MNs (Figures 1G, 1H, and S1A). Thus, a single mutant *FUS* allele was enough to drive MN degeneration. Finally, morphometric analysis of our neuronal cultures revealed that, on average, FUS H517Q/H517Q MN had reduced neurite length compared to the healthy controls and H517Q/+ MN (Figures 1I and S1A). Overall, our *in vitro* iPSC model recapitulated essential characteristics of MN degeneration observed in ALS.

FUS MN display widespread dysregulation of miRNA expression

miRNAs are post-transcriptional regulators of gene expression that suppress the translation of their target mRNAs and can fine-tune signaling pathways (Ha and Kim, 2014). Dysregulation of miRNA expression has been observed in fALS and sALS (Emde et al., 2015; Figueroa-Romero et al., 2016; De Santis et al., 2017; Reichenstein et al., 2019; Tung et al., 2019; Kurita et al., 2020). Hence, we used small RNA sequencing (RNA-seq) to profile miRNA expression levels in our disease model. Principal-component analysis (PCA) and hierarchical clustering indicated that the major source of variation in our data was the underlying sample genotype (Figures 2A and S1B). Sample 79A R1 was an outlier and was excluded from further analysis. DESeq2 analysis identified 81 miRNAs in the FUS H517Q/H517Q MN and 38 miRNAs in the H517Q/+ MN as differentially regulated (adjusted $p < 0.05$, absolute log2 fold change >0.5) (Figure 2B).

Almost equal numbers of miRNAs were upregulated and downregulated (41 up versus 39 down, H517Q/H517Q versus healthy and 15 up versus 23 down, H517Q/+ versus healthy) in FUS ALS MN. These results contrast with miRNA profiling data obtained in sALS MNs, where most miRNAs were observed as downregulated (Emde et al., 2015). One reason for this discrepancy could be that the profiling was performed at different stages. We profiled miRNA expression levels at day 30 before the onset of degenerative phenotypes, while postmortem analysis represents the end stage of the disease. Likely, in the end stages of the disease, miRNA biogenesis is severely impaired, resulting in a global miRNA downregulation.

MiR-139 is downregulated in FUS and sALS MN

We compared our expression data with three miRNA datasets generated from sALS tissue, and our previous study in which we profiled miRNAs dysregulated in FUS P525L MNs (Figure 2C). To identify high-confidence miRNA candidates that are dysregulated in FUS and sALS, we only retained miRNAs that showed a significant expression change in at least three out of the six datasets analyzed.

The overlap of differentially expressed miRNAs between ALS datasets was low, highlighting the well-known polygenic architecture of ALS (Figure 2C). MiR-139-5p, hereafter referred to as miR-139, was the only miRNA that was commonly downregulated across fALS and sALS datasets. Furthermore, miR-139 is downregulated in patient serum and postmortem spinal cord tissue (Raheja et al., 2018; Kurita et al., 2020). qRT-PCR analysis of our iPSC-derived MNs confirmed the downregulation of miR-139 in FUS

(E) Representative images of day 34 MNs differentiated from healthy iPSCs 80a and 79a, FUS H517Q/H517Q, and FUS H517Q/+ ALS iPSCs. MNs were stained for ISL1 and NFH. Nuclei were stained with Hoechst. Scale bar indicates 50 μm.
(F) MN differentiation efficiency, determined by $ISL1^{+}$ nuclei. N = 3–5.
(G) FUS MN ($ISL1^{+}$, $TUJ1^{+}$) display survival loss in culture compared to healthy MN. H517Q/+ MN display improved survival *in vitro*, indicating that survival defect is due to mutation. Days 30 and 45 indicate days 30 and 45 in the differentiation process, with day 0 marking the first media change on the iPSC. N = 3.
(H) FUS ALS MNs show an increased percentage of apoptotic cells. N = 3.
(I) Quantitation of neurite complexity for day 45 MNs. Neurite complexity values in pixels for each genotype were normalized to values obtained for healthy control 79a day 45 MNs. FUS MN display reduced neurite complexity compared to healthy MNs. N = 3. N: number of independent differentiations. Error bars indicate SEMs. *p < 0.05, **p < 0.01.

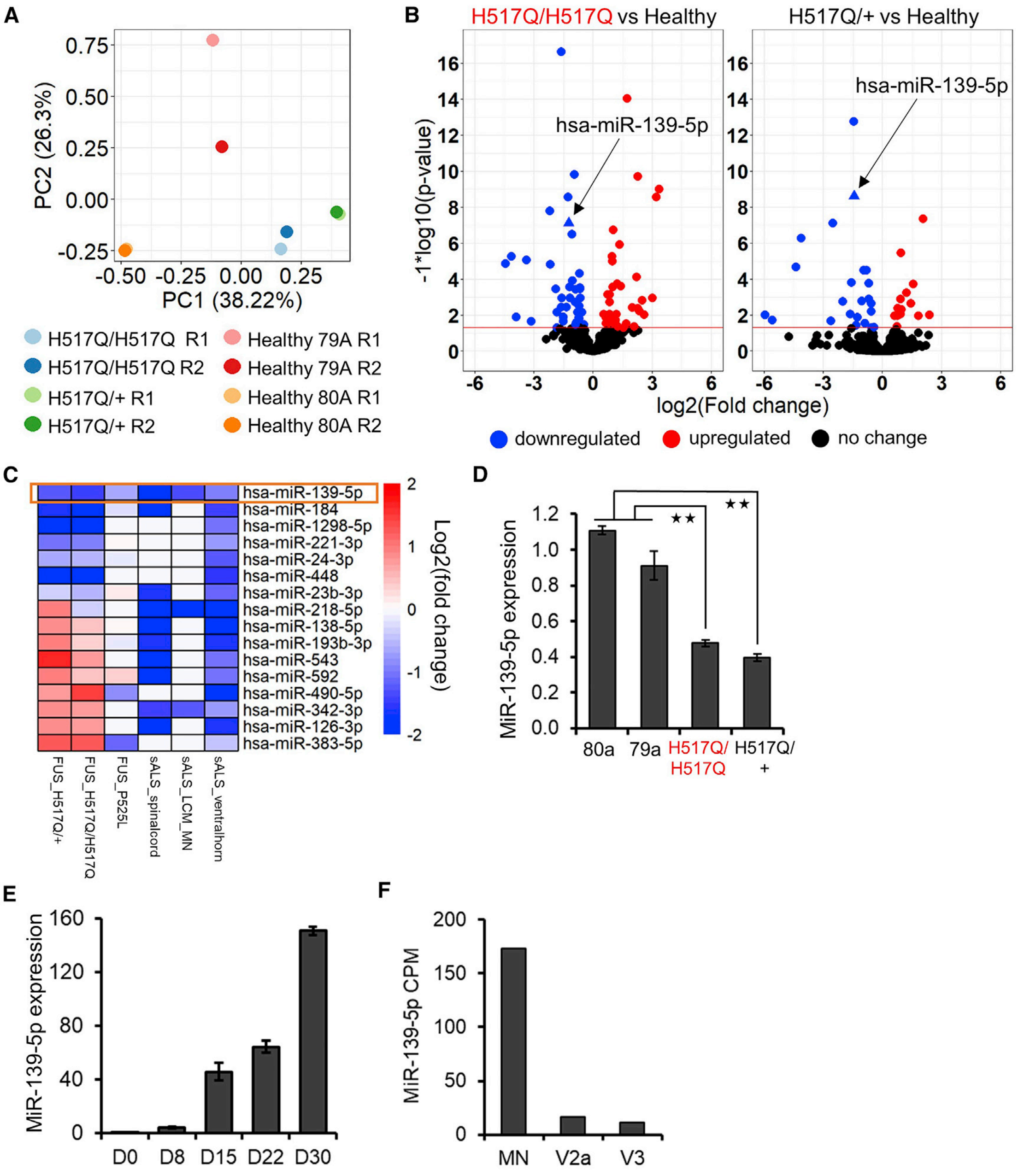

Figure 2. MiR-139-5p is downregulated in ALS MN

(A) Principal-component analysis (PCA) of 844 miRNAs in FUS H517Q/H517Q, FUS H517Q/+ and healthy MN harvested at day 30. N = 2 independent differentiations.

(B) Volcano plot of differentially expressed miRs in H517Q/H517Q versus healthy MNs (left panel) and H517Q/+ versus healthy MNs (right panel). MiR-139 is shown as a triangle (arrow).

(C) Heatmap of miRs identified as differentially expressed in at least 3 ALS datasets out of 6 analyzed. Red indicates upregulation, while blue indicates downregulation. Only miR-139 was commonly downregulated across all datasets (orange rectangle). sALS_spinalcord: postmortem cervical and thoracic spinal cord tissue from ALS patients versus healthy controls (Figueroa-Romero et al., 2016), sALS_LCM_MN: MNs enriched by laser capture microdissection of lumbar spinal cord tissue from ALS patients versus healthy controls (Emde et al., 2015). sALS_ventralhorn: lumbar ventral horn tissue of sporadic ALS versus non-degeneration controls (Reichenstein et al., 2019).

(D) Quantitative RT-PCR (qRT-PCR) confirmation of miR-139-5p downregulation in FUS H517Q MNs. N = 3–5 independent differentiations.

(legend continued on next page)

H517Q/H517Q and H517Q/+ MNs compared to healthy controls (Figure 2D), validating our sequencing results. Next, we profiled the expression of miR-139 as healthy iPSCs differentiated into MNs. Our time course analysis indicated a 150-fold increase in the expression of miR-139 during neuronal differentiation, with the highest abundance observed in mature MNs at day 30 (Figure 2E). Finally, analysis of miR-139 expression in mouse MNs and V2 and V3 interneurons (INs) (Amin et al., 2015) showed that miR-139 is highly enriched in MN compared to neighboring INs (Figure 2F). Taken together, this indicated that miR-139 may play a role in MN specification, maturation, and homeostasis. Hence, we decided to explore the role of miR-139 in human MNs.

MiR-139 is a regulator of WNT signaling

Downstream targets of a miRNA can reveal its functional role within a particular cell type. To determine the downstream targets of miR-139 in MNs, we overexpressed miR-139 in the H517Q/H517Q line and analyzed downstream changes in genome-wide gene expression using RNA-seq. PCA confirmed well-separated experimental conditions, indicating that miR-139 overexpression (OE) induced significant changes in gene expression (Figure 3A). We observed an enrichment of predicted miR-139 targets in the set of genes downregulated by miR-139 OE (Figure 3B). Furthermore, we observed a significant enrichment of miR-139 7mer seed sequences in the downregulated gene set, indicating that our analysis could detect direct miRNA targets (Figure 3C). Gene set enrichment analysis (GSEA) (Subramanian et al., 2005; Liberzon et al., 2011) identified targets of the WNT mediator β-catenin (CTNNB1) in the top 10 gene sets that were enriched in genes downregulated by miR-139 OE (Figure 3D). Analysis of miR-139 predicted targets revealed that several WNT mediators, including β-catenin, are potential direct targets of miR-139 (Figure 3E).

β-Catenin is the major effector/transcriptional mediator of the canonical WNT signaling pathway (Figure S2A). Previous studies have revealed that miR-139 inhibits WNT/β-catenin signaling in immortalized/cancer cells (Anton et al., 2011; Long et al., 2017; Gu et al., 2014). Hence, we explored β-catenin protein abundance and activity after miR-139 modulation in MNs. OE of miR-139 in FUS H517Q/H517Q MN resulted in a 40% reduction in β-catenin protein levels, while mRNA levels were reduced ~20% (Figures 3F, S2B, and S2C). In healthy 80a MNs, miR-139 OE resulted in >50% reduction in β-catenin protein levels (Figures 3F and S2B), indicating that miR-139 can inhibit canonical WNT signaling in MN. These data also reflect that miRNAs may only inhibit translation and not necessarily cause transcript degradation. To confirm that miR-139 targets β-catenin directly, we conducted a 3′ UTR luciferase reporter assay in healthy MNs. Mutation of the predicted miR-139 binding site in the CTNNB1 3′ UTR (identified by Targetscan) resulted in a >3-fold increase in luciferase signal compared to the non-mutated 3′ UTR, indicating that β-catenin is a direct target of miR-139 in human MNs (Figure 3G). Next, we asked whether the inhibition of miR-139 could result in the activation of WNT signaling. Since SH-SY5Y neuroblastoma cells express miR-139 endogenously and are relatively easy to transfect, we used this neural cell line to further investigate the interaction of miR-139 and WNT. Inhibition of miR-139 using antagomirs resulted in a 100% increase in β-catenin activity, as measured by SuperTopFlash reporter luciferase assay (Figure 3H). Interestingly, the stimulation of WNT in SH-SY5Y cells with the agonist CHIR99021 resulted in the upregulation of miR-139 (Figure 3I), revealing a negative feedback loop between WNT and miR-139 (Figure 3J).

Since miR-139 is downregulated in sALS, we hypothesized that WNT pathway genes targeted by miR-139 (miR-139-WNT gene set) would be upregulated in sALS datasets. We observed a significant enrichment of the miR-139-WNT gene set in genes upregulated in sALS MN (Figure 3K), suggesting that disruption of the miR-139-WNT axis may be a common theme across fALS and sALS.

WNT signaling is activated in FUS ALS MNs

As miR-139 is downregulated in ALS MNs and inhibits WNT signaling, we expected WNT signaling to be overactivated in our FUS H517Q MN lines. Accordingly, we found upregulation of several WNT ligands and WNT target genes in the FUS MN by qRT-PCR (Figure 4A). In addition, SuperTopFlash luciferase assays showed an increase in β-catenin activity (Figure 4B), while traditional western blot assays identified an increase in β-catenin protein levels (Figure 4C) in FUS MN compared to healthy MNs. Quantification of β-catenin protein levels, using the highly sensitive and quantitative in-cell western assay, confirmed a greater than 3-fold increase in β-catenin levels in the H517Q/H517Q MNs relative to healthy control MNs (Figures 4D and S3C).

To expand our observations beyond the H517Q FUS mutation, we evaluated WNT activity in our previously

(E) Expression analysis of miR-139-5p over the time course of MN differentiation from healthy iPSCs (day 0). MiR-139-5p levels increase throughout MN differentiation and maturation. N = 3.
(F) MiR-139-5p expression assayed in spinal MN, V2a, and V3 interneurons. CPM indicates counts per million. RNA-seq data were obtained from Amin et al. (2015). Error bars indicate SEMs. **p <0.01 by Student's *t* test.

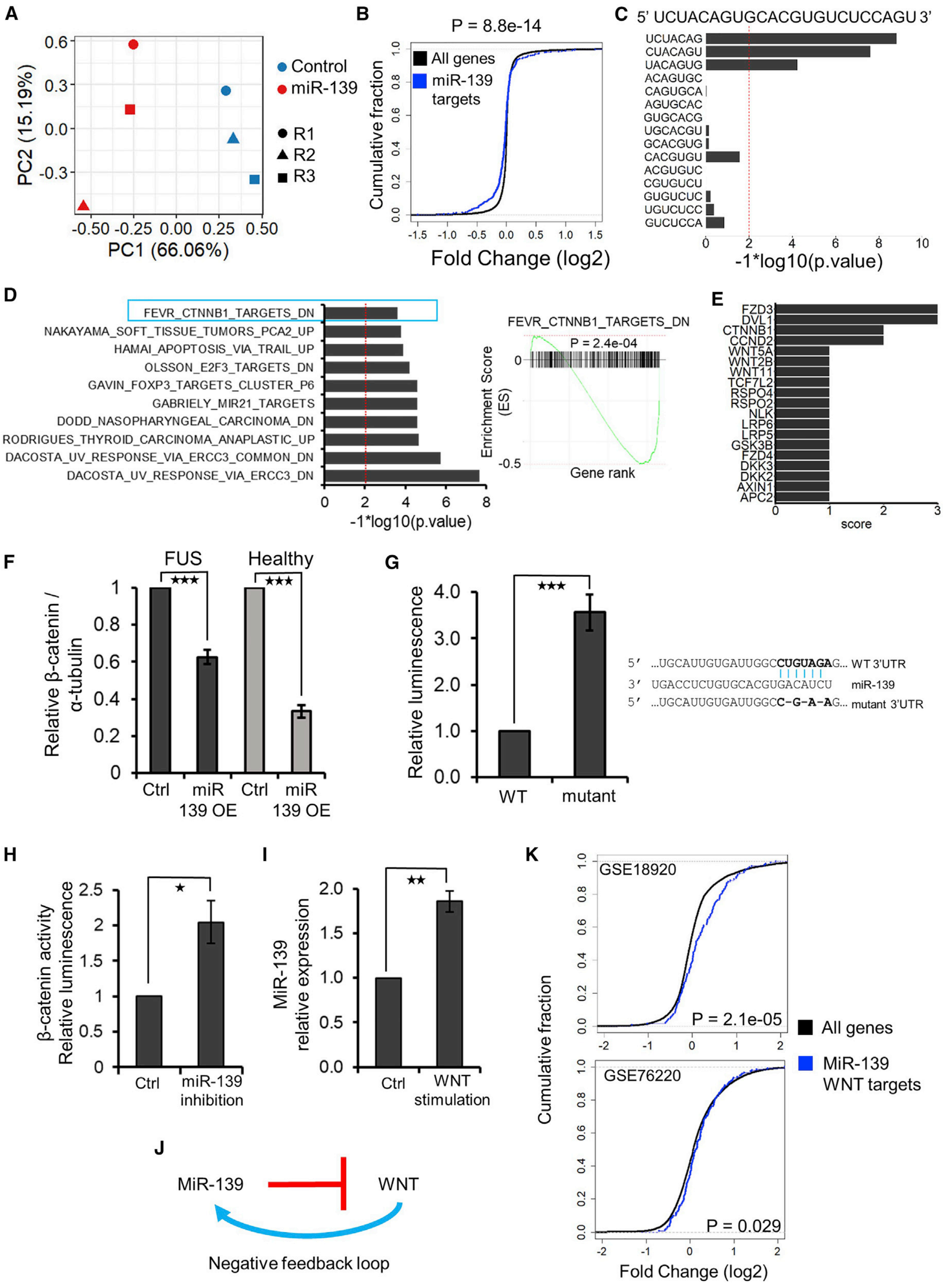

(legend on next page)

characterized FUS P525L iPSC MN (Figure S3A), alongside the appropriate isogenic control (De Santis et al., 2017). In-cell western assays confirmed significant upregulation of β-catenin protein in FUS P525L MNs (Figures 4D and S3C), similar to observations in the FUS H517Q ALS MNs.

Inhibition of canonical WNT signaling improves MN survival

We hypothesized that inhibiting WNT signaling should improve FUS ALS MN survival. We also targeted pathways known to synergistically interact with WNT signaling and activated in ALS, including mitogen-activated protein (MAP) kinase family members p38 (MAPK14), ERK1 and -2, and JNK1, -2, and -3, as well as TP53 (Bhinge et al., 2017; Sama et al., 2017; Gibbs et al., 2018; Červenka et al., 2011; Damalas et al., 1999). Furthermore, we treated cells with a pan-cell-cycle-dependent kinase (CDK) inhibitor as MN death could be due to WNT-driven activation of the cell cycle (Bryja et al., 2017; Frade and Ovejero-Benito, 2015). We used FUS H517Q/H517Q MN as these neurons displayed greater survival deficits than the FUS H517Q/+ MN. FUS ALS MN were treated with small molecule inhibitors of the selected pathways at the indicated concentrations at day 30 and treatment was continued until day 45 (Figures 4E and S4A).

Untreated (vehicle-only control) FUS MN showed a significant loss of survival, with ~50% of the MN lost at day 45 compared to day 30 (Figure 4E). Inhibiting WNT signaling reduced MN loss in a concentration-dependent manner such that only 15% of the MN were lost at day 45 at the highest concentration used (Figure 4E). Inhibition of p38/MAPK signaling similarly restricted FUS MN loss to 25% by day 45 (Figure 4E). Surprisingly, treatment with the ERK and JNK inhibitors had no effect on FUS MN survival (Figure S4A), as opposed to the significant effects observed in SOD1 MN (Bhinge et al., 2017). Inhibition of the TP53 pathway led to a modest but statistically significant increase in FUS MN survival (Figure S4A). Treatment with the pan-CDK inhibitor enhanced MN survival by 12% at the lowest concentration (0.15 μM), but was toxic at higher concentrations (Figure S4A). In summary, our pharmacological inhibitor screen revealed that WNT signaling and p38/MAPK are major drivers of FUS ALS MN loss, similar to observations in SOD1 E100G iPSC-derived MN (Bhinge et al., 2017).

To further validate the role of canonical WNT signaling in MN degeneration, we overexpressed β-catenin in healthy 80A MN and observed a more than 2-fold increase in caspase activity, indicating increased apoptosis (Figures 4F and S4B). Taken together, we conclude that

Figure 3. MiR-139-5p targets WNT signaling in FUS ALS MNs

(A) PCA using the top 500 variable genes from miR-139 overexpression (OE) in FUS H517Q MNs. RNA collected at day 35.

(B) Cumulative distribution function (CDF) plot comparing the fold changes of predicted miR-139 targets (blue curve) relative to all genes (black curve) in response to miR-139 OE. p values were estimated using the Kolmogorov-Smirnov test.

(C) Enrichment of 7mers starting at positions 1–15 of miR-139-5p in the 3′ UTR of genes downregulated upon miR-139-5p OE. The miRNA sequence is shown at top for reference. p values were estimated using a hypergeometric distribution and corrected for multiple hypothesis testing using the Benjamini-Hochberg method. A p value threshold of 0.01 is indicated by the vertical dotted line.

(D) Left panel: GSEA analysis of miR-139 OE using curated gene sets from MSigDB. X axis displays the log transformed p values. Genes downregulated upon *CTNNB1* deletion in intestinal epithelial cells (FEVR_CTNNB1_TARGETS_DN) were identified in the top 10 gene sets with a significant negative enrichment score (i.e., these gene sets were downregulated after miR-139-5p OE). Right panel: GSEA enrichment plot for the gene set FEVR_CTNNB1_TARGETS_DN. X axis shows genes sorted from most upregulated (left-hand side) to most downregulated (right-hand side). Enrichment p values for the gene sets were estimated using 1,000 random simulations and adjusted using the Benjamini-Hochberg method.

(E) MiR-139-5p is predicted to directly target several WNT-associated genes, including the WNT transcriptional mediator CTNNB1. Predicted targets were obtained from 3 databases: miRWalk, Targetscan, and miRDB (Sticht et al., 2018; Agarwal et al., 2015; Wong and Wang, 2015). Score indicates the number of databases supporting each interaction.

(F) In-cell western blot analysis to measure β-catenin protein levels in response to miR-139-5p overexpression (OE) in FUS H517Q/H517Q ALS (N = 5) and healthy 80a MNs (N = 3).

(G) Relative luminescence of the Renilla luciferase gene, carrying the CTNNB1 3′ UTR with the predicted miR-139 binding site (wild type [WT]) or with the mutated site (mutant). Mutation of the miRNA binding site rescues luciferase activity when transfected in human MNs. N = 5.

(H) SuperTopFlash dual luciferase assay to measure β-catenin activity upon inhibition of miR-139 in SH-SY5Y cells. N = 3.

(I) qRT-PCR analysis of miR-139-5p expression upon activation of WNT signaling using CHIR09921 in SH-SY5Y cells. N = 3.

(J) Schematic of proposed negative feedback loop between miR-139 and the WNT pathway.

(K) CDF plot of miR-139-5p WNT targets (blue) and all expressed genes (black) in differentially expressed genes in laser capture microdissected sALS MN relative to healthy controls. GSE18920: MNs were derived from lumbar spinal cords (12 ALS and 10 controls) and gene expression analyzed using microarrays. GSE76220: MN were derived from lumbar spinal cords (13 ALS and 8 controls) and gene expression analyzed by RNA-seq. The p value was calculated using the Kolmogorov-Smirnov test. N: number of independent differentiations. Error bars indicate SEMs. *$p < 0.05$, **$p < 0.01$, ***$p < 0.001$ by Student's *t* test, unless otherwise stated.

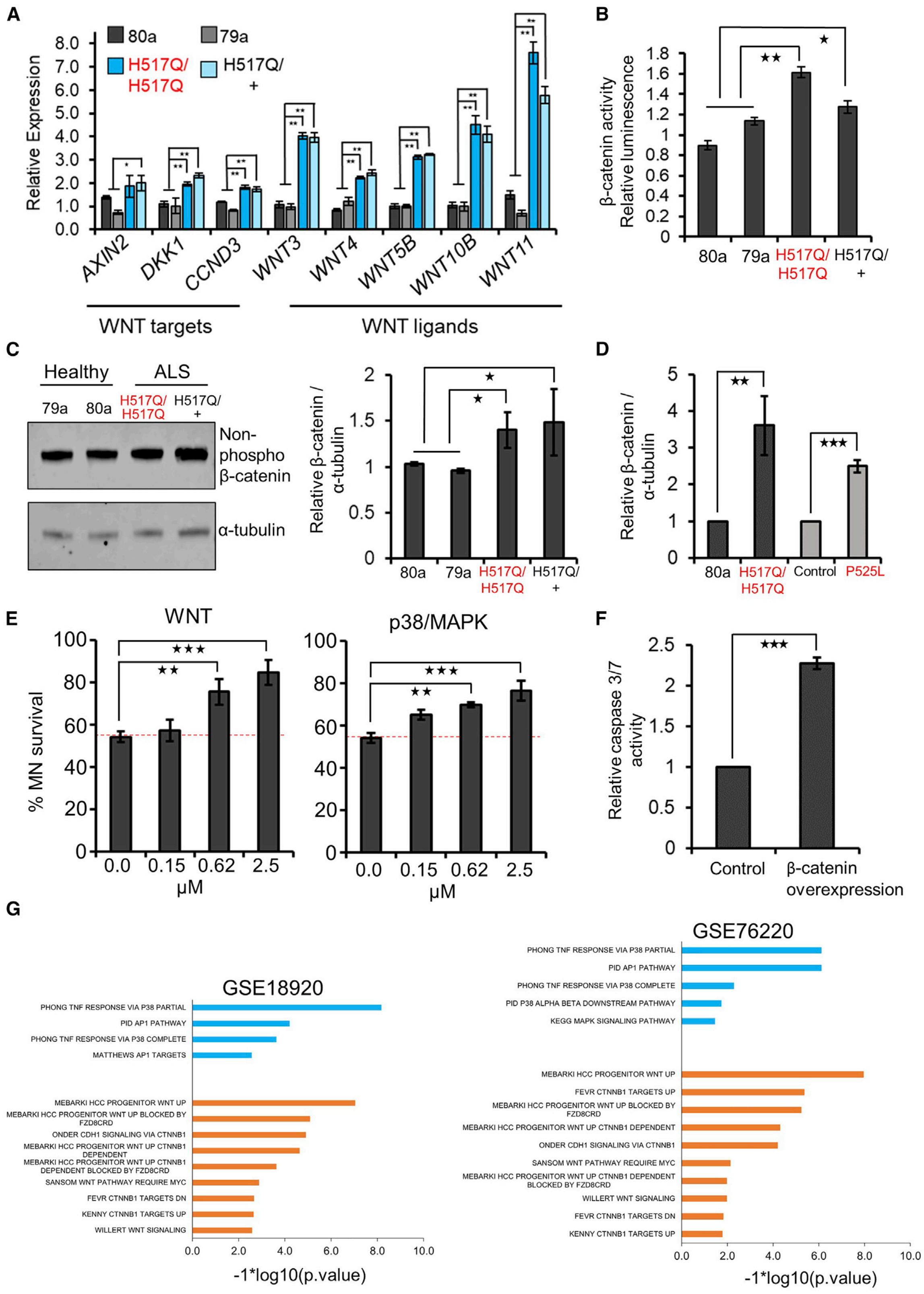

(legend on next page)

the upregulation of WNT alone is enough to drive degeneration in iPSC-derived MNs.

To investigate the relevance of the identified pathways to sALS, we performed GSEA on the sALS MN datasets using gene sets related to WNT/β-catenin or p38/MAPK perturbation. Enrichment analysis showed significant upregulation of several WNT and p38/MAPK gene sets in sALS MNs (Figure 4G). Overall, our results indicate that the hyperactivation of WNT signaling, at least partly due to the downregulation of miR-139, drives MN degeneration in both fALS and sALS MN. Interestingly, miR-139 inhibition has been shown to activate p38/MAPK signaling in colon tumor tissue (Zou et al., 2016). It is likely that the downregulation of miR-139-5p also leads to activation of p38/MAPK in MNs.

Mutant FUS downregulates miR-139 potentially by a gain-of-function mechanism

FUS and TDP-43 are involved in different stages of the miRNA biogenesis pathway (Butti and Patten, 2018). To assess whether FUS influences miR-139 through a loss of function, we depleted FUS in SH-SY5Y cells and measured miR-139 expression by qRT-PCR (Figures 5A and 5B). Although we could deplete FUS by 85% using small interfering RNA (siRNA) (Figure 5A), the observed downregulation of miR-139 was less than 20% (Figure 5B). Depletion of FUS in healthy MN progenitors for 3 days failed to produce any change in miR-139 levels (Figure 5B). This suggested that miR-139 downregulation was not due to a loss of function of FUS, supporting recent work indicating that FUS-associated ALS is due to toxic gain of function (Korobeynikov et al., 2022; Sharma et al., 2016). As FUS has been shown to regulate RNA polymerase II (RNAPII)-mediated transcription (Schwartz et al., 2012), we hypothesized that mutant FUS may affect miR-139 at the transcriptional level.

We quantified primary miRNA (pri-miRNA) levels from RNA-seq data by measuring intronic reads mapping to host genes (supplemental experimental procedures) (Gaidatzis et al., 2015). We tested the feasibility of this approach using our RNA-seq data for the FUS P525L MN. Here, we had previously analyzed expression levels of protein-coding genes and miRNAs in parallel (De Santis et al., 2017). We used the MiRIAD database (Hinske et al., 2017) to identify intronic miRNAs and their corresponding host genes expressed in the FUS P525L or isogenic MN (Figure 5C). Next, we plotted fold changes in the mature miRNAs expression against the fold changes of the intronic read counts of their host genes. We identified a modest but significant correlation between the miRNAs and their host gene intron expression (Figure 5D), suggesting that the expression level of mature miRNAs was largely due to transcriptional control. Example exceptions to this statement are indicated in Figure 5D by a blue asterisk.

Our analysis showed expression changes in the mature as well as the pri-miRNA transcript of miR-139 (Figure 5D, arrow). MiR-139 is located within an intronic region of the *PDE2A* gene (Kent et al., 2002). Analysis of intronic reads mapping to *PDE2A* showed that pri-miR-139 transcript levels were reduced by 30% in the FUS P525L MN compared to the isogenic controls (Figure 5E). This was similar to the 35% reduction seen for the mature miR-139 in the FUS P525L MN (Figure 5D). Intronic miRNAs 185, 22, and 26a, which were identified as unchanged in our iPSC MN model did not display any changes in their pri-miRNA levels, as expected (Figure 5E).

Next, we conducted deep RNA-seq on ribosomal depleted total RNA for the FUS H517Q/H517Q and healthy 80A MNs to obtain better coverage of reads mapping to intronic regions of the genome. As observed in the P525L dataset, we identified a significant correlation between changes in expression of the mature miRNAs and their corresponding

Figure 4. ALS MNs display increased WNT

(A) qRT-PCR analysis of mRNA expression of WNT genes and ligands in FUS ALS MNs compared to healthy controls (79a and 80a). N = 3.

(B) SuperTopFlash luciferase assay showing relative luciferase units for β-catenin activity in H517Q/H517Q and H517Q/+ MN relative to 80a and 79a healthy controls. N = 3–4.

(C) Representative western blot (left panel) and quantification (right panel) of active β-catenin protein in healthy and FUS ALS MNs. β-catenin normalized to α-tubulin and shown relative to healthy controls. N = 3.

(D) In-cell protein quantification of β-catenin in FUS H517Q/H517Q MN relative to healthy control 80a (N = 5) and FUS P525L MN relative to isogenic control (N = 3).

(E) Quantification of $ISL1^{+}$ FUS H517Q/H517Q MNs at day 45 relative to day 30 after treatment with small-molecule inhibitors of the indicated pathways. Horizontal dotted line indicates MN loss in the DMSO-only control. N = 3, p values were estimated using Student's 2-tailed t test.

(F) Caspase-3/7 activity relative to viable cells in response to overexpression of β-catenin versus GFP control in day 23 healthy (80A) MNs. N = 3.

(G) GSEA on sALS MN datasets using gene sets relevant to WNT or p38/MAPK signaling. Blue bars indicate p38/MAPK gene sets. Orange bars indicate WNT/CTNNB1 gene sets. p values were estimated using 1,000 random simulations and adjusted using the Benjamini-Hochberg procedure. N: number of independent differentiations. Error bars indicate SEMs. p values were estimated by Student's 1-tailed t test unless otherwise indicated. *p < 0.05, **p < 0.01, ***p < 0.001.

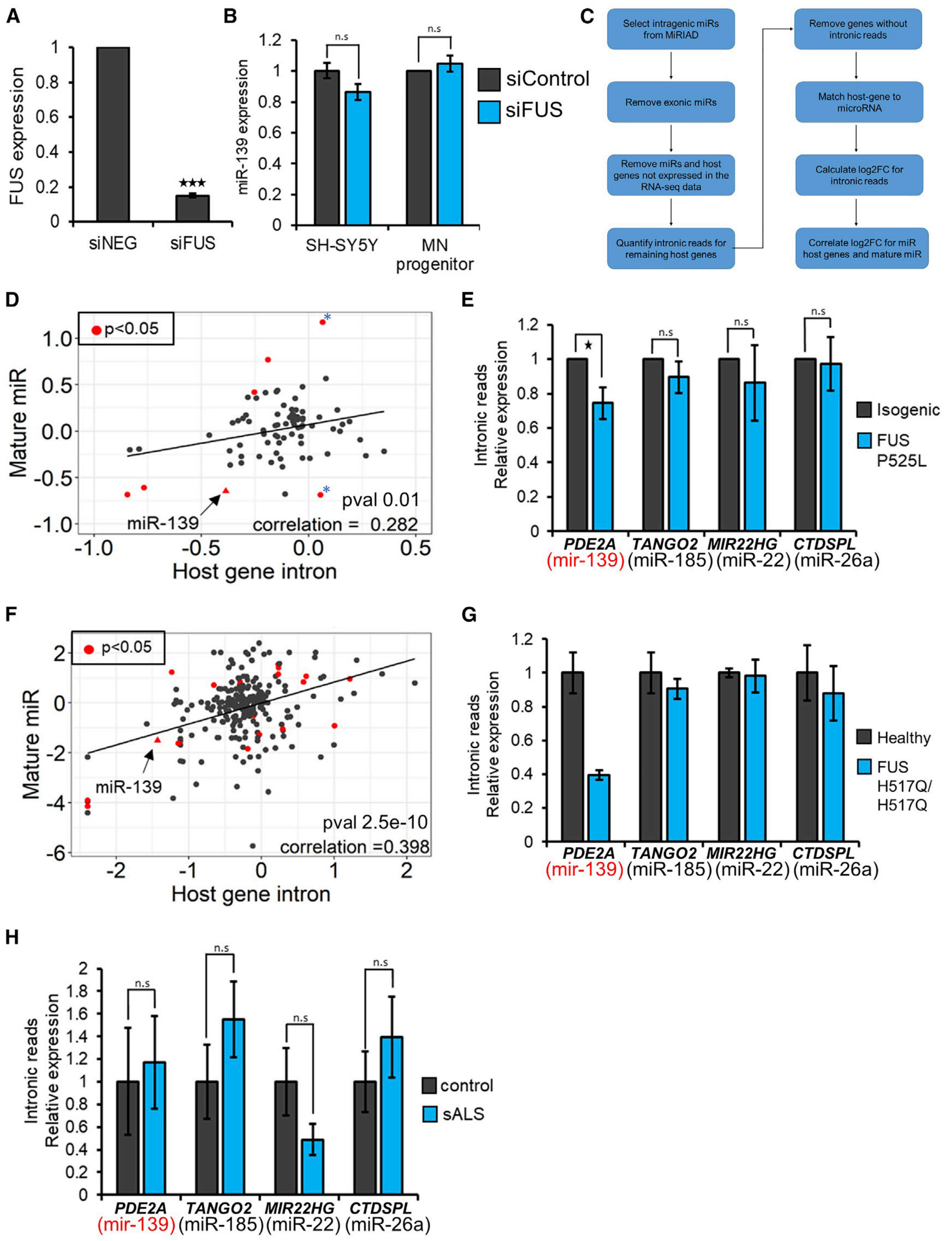

Figure 5. Mutant FUS transcriptionally downregulates miR-139 potentially through a gain-of-function mechanism

(A) Relative FUS gene expression after siRNA-mediated knockdown in SH-SY5Y cells. Fold changes were normalized to scrambled (negative control) siRNA. N = 3 independent transfections. p values were estimated using Student's 1-tailed t test.

(B) qRT-PCR analysis of miR-139-5p expression in response to siRNA-mediated FUS knockdown in SH-SY5Y cells and MN progenitors derived from healthy (80a) iPSC. Gray bars: scrambled siRNA used as a negative control; blue bars: siRNA against FUS. N = 3. Fold changes were normalized to scrambled siRNA.

(legend continued on next page)

host genes (Figure 5F). We observed a 60% reduction in intronic reads of *PDE2A* in FUS H517Q/H517Q MN (Figure 5G), with the reduction proportional to that seen for the mature miR (Figures 2B and 5F). Our results indicate that the observed changes in mature miR-139 levels are at least partly due to the corresponding downregulation of the primary miR-139 transcript.

These results demonstrate the potential of using deep RNA-seq data to predict transcriptional changes in pri-miRNAs and indicate that mutant FUS may impede transcription of the pri-miR-139 transcript. However, when we performed a similar analysis on RNA-seq data obtained from sALS MNs (GSE76220), we did not observe any decrease in the intronic reads of *PDE2A*. Our analysis suggests that downregulation of pri-miR-139 transcription could be specific to FUS mutations only.

DISCUSSION

It is unclear whether the widespread downregulation of miRNAs observed in ALS MN is an effect of dying neurons or if it contributes to neuronal death. In addition, the differential susceptibility of MN in ALS may be due to genes that are differentially active in MNs, but are disrupted in disease. We observed that miR-139 was activated during MN differentiation, is highly expressed and enriched in mature MN, and was downregulated in both mutant FUS and sALS MN. This suggested that the downregulation of miR-139 in MNs can lead to the disruption of homeostatic mechanisms. We found that miR-139 inhibits the canonical WNT pathway in human MNs through the direct targeting of β-catenin, and is reciprocally activated by an increase in WNT signaling. This negative feedback loop between miR-139 and WNT may be required to keep WNT activity within a desired range in MNs.

Dysregulation of WNT, either overactivation or inhibition, has been implicated in causing neurodegeneration in adults. Decrease in canonical WNT signaling has been linked to Alzheimer's disease (AD), in which inhibition of canonical WNT and activation of the non-canonical WNT/PCP leads to synaptic disassembly (Purro et al., 2014; Sellers et al., 2018). However, the upregulation of WNT pathway components has been associated with Huntington's disease (HD) and ALS. For example, in Drosophila neurons, expression of mutant huntingtin protein stabilizes β-catenin and leads to neuronal death, which can be reversed by the RNAi-mediated knockdown of β-catenin (Godin et al., 2010). In ALS, increased levels of WNT ligands, receptors, and downstream targets have been identified in human postmortem tissue (spinal cord, anterior horn) and SOD1G93A transgenic mouse models (spinal cord) (Yu et al., 2013; Chen et al., 2012a, 2012b; González-Fernández et al., 2019).

ALS-associated mutant Ubiquilin 4 (UBQLN4) has been linked to the upregulation of β-catenin when expressed in mouse spinal MN *in vitro* and zebrafish MN *in vivo* (Edens et al., 2017). Pharmacological inhibition of the nuclear localization of β-catenin rescued axonal defects observed in these models (Edens et al., 2017). β -Catenin was also identified as a key driver of neurodegeneration in animal models of spinal muscular atrophy (SMA), an MN disease affecting infants and children (Wishart et al., 2014). These observations indicate that the upregulation of WNT/β-catenin signaling can be detrimental to MN survival and homeostasis. WNT has been shown to be upregulated through autocrine mechanisms in cancer cell models (Bafico et al., 2004; Schlange et al., 2007), as well as neuronal cell populations (Wexler et al., 2009; Ryu et al., 2013). Our data suggest that miR-139 may also regulate WNT signaling through targeting WNT ligands, in addition to inhibiting β-catenin, to break the autocrine loop. Overall, our results indicate that WNT activity is tightly controlled in human MN to

(C) Flowchart depicting the correlation analysis between expression changes in intronic miRs and their host genes.
(D) Scatterplot contrasting log2 fold changes in intronic reads of a host gene versus the corresponding mature miR in FUS P525L MNs relative to the isogenic control MNs. Number of miRs = 80, N = 3. Shown in red are miRNAs identified as differentially expressed in FUS P525L MN at day 19 ($p < 0.05$, data from our previous publication; De Santis et al., 2017). Blue asterisks indicate example miRNAs described in Mutant FUS downregulates miR-139 potentially by a gain-of-function mechanism.
(E) Relative expression of pri-miRNAs for miR-139-5p, miR-185, miR-22 and miR-26a in FUS P525L MNs. Intronic counts for the isogenic sample were set to 1. N = 3.
(F) Scatterplot contrasting log2 fold changes in intronic reads of host genes versus the corresponding mature miRs in FUS H517Q MNs relative to the healthy control MNs. Number of miRs = 208, N = 2. Shown in red are miRNAs identified as differentially expressed in FUS H517Q MN at day 30 (adjusted $p < 0.05$).
(G) Relative expression of pri-miRNAs for miR-139-5p, miR-185, miR-22, and miR-26a in H517Q MNs. Intronic counts for the isogenic sample were set to 1. N = 2. Adjusted p value for PDE2A/miR-139 = 3.8e−10 estimated using DESeq2 analysis.
(H) Relative expression of pri-miRNAs for miR-139-5p, miR-185, miR-22, and miR-26a in sALS MNs. Intronic counts for the control MNs were set to 1. N = 13 for ALS and N = 8 for controls. N: number of independent differentiations. Error bars indicate SEMs. *$p < 0.05$, **$p < 0.01$, ***$p < 0.001$.

maintain homeostasis and prevent neurodegeneration. Expression of miR-139 is one of the mechanisms involved in this regulation. It is unlikely that miR-139 is the sole miRNA responsible for regulating WNT activity in MN. Indeed, other miRNAs implicated in regulating WNT signaling have been observed to be downregulated in ALS MNs. For example, miR-375 targets the canonical WNT pathway and is downregulated in FUS P525L MN (Wang et al., 2013; De Santis et al., 2017). MiR-218 is the most significantly downregulated miRNA in sALS MNs and has been shown to inhibit as well as promote WNT signaling (Reichenstein et al., 2019; Tu et al., 2013; Anton et al., 2011; Zhao et al., 2019). Consequently, WNT signaling is under the control of multiple miRNAs, and the upregulation of WNT activity in ALS MNs is likely due to the dysregulation of multiple WNT-regulating miRNAs.

How does WNT hyperactivation lead to MN death? WNT signaling is typically implicated in cell growth, proliferation, and specification (Clevers, 2006). Previous studies have observed an upregulation in the cell cycle in ALS MNs and inhibition of CDKs improve disease phenotypes (Bhinge et al., 2017; Rojas-Prats et al., 2021; Ranganathan and Bowser, 2010). An attractive possibility is that cell-cycle reactivation through aberrant WNT signaling contributes to MN death (Frade and Ovejero-Benito, 2015; Bryja et al., 2017). However, inhibition of the cell cycle by a pan-CDK inhibitor led to a modest increase in MN survival and was toxic at higher doses. It is possible that some CDKs have a critical cell-cycle-independent role to play in MN homeostasis. In this case, targeting specific CDKs may improve ALS MN survival as observed in SMA (Hor et al., 2018) and TDP43 mutant mice (Rojas-Prats et al., 2021).

A related question is what causes miR-139 downregulation in ALS MNs. FUS-ALS accounts for only a small proportion of ALS cases, yet we also see miR-139 downregulated in sALS without FUS or TDP-43 mutation. However, defects in TDP43 and FUS localization are observed in sALS, while both proteins regulate aspects of miRNA biogenesis (Morlando et al., 2012; Kawahara and Mieda-Sato, 2012; Zhang et al., 2018; Ling et al., 2010). Hence, mislocalization of these RNA-binding proteins may cause defects in miRNA biogenesis. Our findings raise the possibility that FUS may also affect the transcription of ALS-associated miRNAs. We observed a higher absolute fold change for the mature miR-139 expression in comparison to the intronic *PDE2A* levels, for both H517Q and P525L datasets. Therefore, in FUS-ALS, the downregulation of miR-139 could be due to a combination of reduced pri-miR-139 transcription and downstream miRNA processing defects. However, in sALS, we could not detect significant changes in the miR-139 pri-miRNA, suggesting that defects in miRNA processing are likely to be the primary cause of miR-139 downregulation.

Overall, our results highlight the importance of RNA processing in ALS and demonstrate the knock-on effect a single miRNA can have on transcriptomic equilibrium that can disrupt MN homeostasis.

EXPERIMENTAL PROCEDURES

miRNA overexpression

Adeno-associated viruses (AAVs) expressing miR-139 or small hairpin RNA (shRNA) against *OCT4* were generated using the AAVpro Helper Free System (Takara). *OCT4* is not expressed in MN; hence, this shRNA was used as a non-targeting control. The mature miR-139-5p sequence was expressed using a shRNA design to ensure the expression of miR-139-5p and not miR-139-3p (Brummelkamp et al., 2002; Qu et al., 2012) (Figure S5). MNs were transduced at day 23 and collected for RNA at day 34.

miRNA inhibition

SH-SY5Y cells were plated in 96-well optically clear white tissue culture plates (Greiner) 24 h before transfection with a negative or miR-139-5p inhibitor (IDT) using lipofectamine STEM reagent (Thermo Fisher Scientific). Inhibitors were used at a final concentration of 20 nM. For luciferase assay, inhibitors were cotransfected with 50 ng STF19 plasmid (Psicheck2, Promega) modified to contain 19 binding sites for β-catenin/TCF upstream of the firefly luciferase gene, and 5 ng Renilla luciferase plasmid (pRLcmv, Promega) as an internal control. Media was replaced 24 h post-transfection and the luciferase assay completed 72 h post-transfection as per protocol (Dual-Glo Luciferase Assay System [Promega]). Readings for Firefly and Renilla luciferase were taken on the CLARIOstar Plus microplate reader (BMG Labtech).

SuperTopFlash luciferase assays

Day 30 MNs were plated in 96-well optically clear white tissue culture plates (Greiner). Cells were transfected with 700 ng STF19 plasmid (Psicheck2, Promega) and 100 ng Renilla luciferase plasmid (pRLcmv, Promega). Cells were transfected using a calcium-phosphate method modified from Jiang and Cheng (2006). A final concentration of 12.4 mM $CaCl_2$ was used per well, and transfection mixes were 1/10 of the volume covering cells. Plasmid and $CaCl_2$ master mixes were mixed gradually into Hank's balanced salt solution (HBSS) to prevent the formation of large transfection complexes, and incubated for 20 min at room temperature before addition to wells. Cells were incubated with transfection mixtures at 37°C, 5% CO_2 for 4.5 h, then washed once with 300 mM sodium acetate dissolved in DMEM:F12 medium for 5 min, 37°C to dissolve transfection complexes. Cultures were maintained in neuronal differentiation media supplemented with brain-derived neurotrophic factor (BDNF) and glial cell line-derived neurotrophic factor (GDNF) for a further 72 h, then lysed according to the Dual-Glo Luciferase Assay System (Promega).

UTR luciferase assay

The miR-139 binding site in the 3′ UTR of the CTNNB1 transcript was identified using Targetscan (Agarwal et al., 2015) and extended by 100 bp either side of the predicted site. The resultant sequence

was cloned downstream of the Renilla luciferase gene in the PSICheck2 vector (Promega C8021). To mutate the miR-139 binding site, we deleted positions 2, 4, and 6 of the predicted miRNA binding site. Day 30 healthy MNs (80A) were transfected using the same method described in MN SuperTopFlash luciferase assays. MNs were collected 72 h post-transfection and processed using the Dual-Glo Luciferase Assay System (Promega).

β-catenin overexpression

AAVs expressing β-catenin-EGFP or EGFP only were generated using the AAVpro Helper Free System (Takara). β-Catenin-EGFP plasmid was a gift from Alpha Yap (Addgene plasmid #71367) (Han et al., 2014). Healthy MN progenitor cells were transduced at day 9 before notch inhibition. MNs were matured for 2 weeks before harvesting at day 23 for caspase/cell titer assays. Longer cultures were not viable due to cell death caused by β-catenin overexpression.

Data and code availability

The accession number for the sequencing data reported in this paper is GEO: GSE203173.

SUPPLEMENTAL INFORMATION

Supplemental information can be found online at https://doi.org/10.1016/j.stemcr.2022.05.019.

AUTHOR CONTRIBUTIONS

S.H., S.C.N., A.T., C.B., and A.B. designed and performed the experiments and analyzed the data. S.H. analyzed the RNA-seq and microarray data. P.H. analyzed the microarray data. C.F. and N.S.D. helped with imaging. A. Randall performed the Ca^{2+} imaging assays. A. Rosa provided the FUS P525L iPSC lines. A.B. and L.W.S. funded the project. A.B. conceptualized the project. S.H. and A.B. wrote the manuscript. All of the authors contributed toward editing the manuscript.

ACKNOWLEDGMENTS

We thank the sequencing center at the Genome Institute of Singapore and the Exeter sequencing center for help with Illumina sequencing and mapping. We also thank Pia Leete, Jessica Chaffey, and Sarah Richardson for their help with imaging. This work was generously supported by the Wellcome Trust Institutional Strategic Support Award (WT204909MA), the Joint Council Office ASTAR, Singapore, and start-up funds provided to A.B. by the University of Exeter. This work was also supported by an Academy of Medical Science award and an MRC New Investigator Research Grant to A.B.

CONFLICTS OF INTERESTS

The authors declare no competing interests.

Received: July 1, 2021
Revised: May 25, 2022
Accepted: May 26, 2022
Published: June 23, 2022

REFERENCES

Agarwal, V., Bell, G.W., Nam, J.-W., and Bartel, D.P. (2015). Predicting effective microRNA target sites in mammalian mRNAs. Elife *4*, e05005. https://doi.org/10.7554/elife.05005.

Amin, N.D., Bai, G., Klug, J.R., Bonanomi, D., Pankratz, M.T., Gifford, W.D., Hinckley, C.A., Sternfeld, M.J., Driscoll, S.P., Dominguez, B., et al. (2015). Loss of motoneuron-specific microRNA-218 causes systemic neuromuscular failure. Science *350*, 1525–1529. https://doi.org/10.1126/science.aad2509.

Anton, R., Chatterjee, S.S., Simundza, J., Cowin, P., and Dasgupta, R. (2011). A systematic screen for micro-RNAs regulating the canonical Wnt pathway. PLoS One *6*, e26257. https://doi.org/10.1371/journal.pone.0026257.

Bafico, A., Liu, G., Goldin, L., Harris, V., and Aaronson, S.A. (2004). An autocrine mechanism for constitutive Wnt pathway activation in human cancer cells. Cancer Cell *6*, 497–506. https://doi.org/10.1016/j.ccr.2004.09.032.

Bhinge, A., Namboori, S.C., Zhang, X., VanDongen, A.M.J., and Stanton, L.W. (2017). Genetic correction of SOD1 mutant iPSCs reveals ERK and JNK activated AP1 as a driver of neurodegeneration in amyotrophic lateral sclerosis. Stem Cell Rep. *8*, 856–869. https://doi.org/10.1016/j.stemcr.2017.02.019.

Brown, R.H., and Al-Chalabi, A. (2017). Amyotrophic lateral sclerosis. N. Engl. J. Med. *377*, 162–172. https://doi.org/10.1056/nejmra1603471.

Brummelkamp, T.R., Bernards, R., and Agami, R. (2002). A system for stable expression of short interfering RNAs in mammalian cells. Science *296*, 550–553. https://doi.org/10.1126/science.1068999.

Bryja, V., Červenka, I., Čajánek, L., and Čajánek, L. (2017). The connections of Wnt pathway components with cell cycle and centrosome: side effects or a hidden logic? Crit. Rev. Biochem. Mol. Biol. *52*, 614–637. https://doi.org/10.1080/10409238.2017.1350135.

Butti, Z., and Patten, S.A. (2018). RNA dysregulation in amyotrophic lateral sclerosis. Front. Genet. *9*, 712. https://doi.org/10.3389/fgene.2018.00712.

Červenka, I., Wolf, J., Mašek, J., Krejci, P., Wilcox, W.R., Kozubík, A., Schulte, G., Gutkind, J.S., and Bryja, V. (2011). Mitogen-activated protein kinases promote WNT/β-Catenin signaling via phosphorylation of LRP6. Mol. Cell Biol. *31*, 179–189. https://doi.org/10.1128/mcb.00550-10.

Chen, Y., Guan, Y., Liu, H., Wu, X., Yu, L., Wang, S., Zhao, C., Du, H., and Wang, X. (2012a). Activation of the Wnt/β-catenin signaling pathway is associated with glial proliferation in the adult spinal cord of ALS transgenic mice. Biochem. Biophys. Res. Commun. *420*, 397–403. https://doi.org/10.1016/j.bbrc.2012.03.006.

Chen, Y., Guan, Y., Zhang, Z., Liu, H., Wang, S., Yu, L., Wu, X., and Wang, X. (2012b). Wnt signaling pathway is involved in the pathogenesis of amyotrophic lateral sclerosis in adult transgenic mice. Neurol. Res. *34*, 390–399. https://doi.org/10.1179/1743132812y.0000000027.

Clevers, H. (2006). Wnt/β-Catenin signaling in development and disease. Cell *127*, 469–480. https://doi.org/10.1016/j.cell.2006.10.018.

Damalas, A., Ben-Ze'ev, A., Simcha, I., Shtutman, M., Leal, J.F., Zhurinsky, J., Geiger, B., and Oren, M. (1999). Excess beta-catenin promotes accumulation of transcriptionally active p53. EMBO J. *18*, 3054–3063. https://doi.org/10.1093/emboj/18.11.3054.

Deng, H.-X., Zhai, H., Bigio, E.H., Yan, J., Fecto, F., Ajroud, K., Mishra, M., Ajroud-Driss, S., Heller, S., Sufit, R., et al. (2010). FUS-immunoreactive inclusions are a common feature in sporadic and non-SOD1 familial amyotrophic lateral sclerosis. Ann. Neurol. *67*, 739–748. https://doi.org/10.1002/ana.22051.

De Santis, R., Santini, L., Colantoni, A., Peruzzi, G., de Turris, V., Alfano, V., Bozzoni, I., and Rosa, A. (2017). FUS mutant human motoneurons display altered transcriptome and microRNA pathways with implications for ALS pathogenesis. Stem Cell Rep. *9*, 1450–1462. https://doi.org/10.1016/j.stemcr.2017.09.004.

Edens, B.M., Yan, J., Miller, N., Deng, H.-X., Siddique, T., and Ma, Y.C. (2017). A novel ALS-associated variant in UBQLN4 regulates motor axon morphogenesis. Elife *6*, e25453. https://doi.org/10.7554/elife.25453.

Emde, A., Eitan, C., Liou, L.-L., Libby, R.T., Rivkin, N., Magen, I., Reichenstein, I., Oppenheim, H., Eilam, R., Silvestroni, A., et al. (2015). Dysregulated miRNA biogenesis downstream of cellular stress and ALS-causing mutations: a new mechanism for ALS. EMBO J. *34*, 2633–2651. https://doi.org/10.15252/embj.201490493.

Figueroa-Romero, C., Hur, J., Lunn, J.S., Paez-Colasante, X., Bender, D.E., Yung, R., Sakowski, S.A., and Feldman, E.L. (2016). Expression of microRNAs in human post-mortem amyotrophic lateral sclerosis spinal cords provides insight into disease mechanisms. Mol. Cell. Neurosci. *71*, 34–45. https://doi.org/10.1016/j.mcn.2015.12.008.

Frade, J.M., and Ovejero-Benito, M.C. (2015). Neuronal cell cycle: the neuron itself and its circumstances. Cell Cycle *14*, 712–720. https://doi.org/10.1080/15384101.2015.1004937.

Gaidatzis, D., Burger, L., Florescu, M., and Stadler, M.B. (2015). Analysis of intronic and exonic reads in RNA-seq data characterizes transcriptional and post-transcriptional regulation. Nat. Biotechnol. *33*, 722–729. https://doi.org/10.1038/nbt.3269.

Gibbs, K.L., Kalmar, B., Rhymes, E.R., Fellows, A.D., Ahmed, M., Whiting, P., Davies, C.H., Greensmith, L., and Schiavo, G. (2018). Inhibiting p38 MAPK alpha rescues axonal retrograde transport defects in a mouse model of ALS. Cell Death Dis. *9*, 596. https://doi.org/10.1038/s41419-018-0624-8.

Godin, J.D., Poizat, G., Hickey, M.A., Maschat, F., and Humbert, S. (2010). Mutant huntingtin-impaired degradation of beta-catenin causes neurotoxicity in Huntington's disease. EMBO J. *29*, 2433–2445. https://doi.org/10.1038/emboj.2010.117.

González-Fernández, C., Gonzalez, P., Andres-Benito, P., Ferrer, I., and Rodríguez, F.J. (2019). Wnt signaling alterations in the human spinal cord of amyotrophic lateral sclerosis cases: spotlight on Fz2 and Wnt5a. Mol. Neurobiol. *56*, 6777–6791. https://doi.org/10.1007/s12035-019-1547-9.

Gu, W., Li, X., and Wang, J. (2014). miR-139 regulates the proliferation and invasion of hepatocellular carcinoma through the WNT/TCF-4 pathway. Oncol. Rep. *31*, 397–404. https://doi.org/10.3892/or.2013.2831.

Ha, M., and Kim, V.N. (2014). Regulation of microRNA biogenesis. Nat. Rev. Mol. Cell Biol. *15*, 509–524. https://doi.org/10.1038/nrm3838.

Han, S.P., Gambin, Y., Gomez, G.A., Verma, S., Giles, N., Michael, M., Wu, S.K., Guo, Z., Johnston, W., Sierecki, E., et al. (2014). Cortactin scaffolds Arp2/3 and WAVE2 at the epithelial zonula adherens. J. Biol. Chem. *289*, 7764–7775. https://doi.org/10.1074/jbc.m113.544478.

Hawrot, J., Imhof, S., and Wainger, B.J. (2020). Modeling cell-autonomous motor neuron phenotypes in ALS using iPSCs. Neurobiol. Dis. *134*, 104680. https://doi.org/10.1016/j.nbd.2019.104680.

Hinske, L.C., Dos Santos, F.R.C., Ohara, D.T., Ohno-Machado, L., Kreth, S., and Galante, P.A.F. (2017). MiRIAD update: using alternative polyadenylation, protein interaction network analysis and additional species to enhance exploration of the role of intragenic miRNAs and their host genes. Database *2017*, bax053. https://doi.org/10.1093/database/bax053.

Hor, J.H., Soh, E.S.-Y., Tan, L.Y., Lim, V.J.W., Santosa, M.M., Winanto, Ho, B.X., Fan, Y., Soh, B.-S., and Ng, S.-Y. (2018). Cell cycle inhibitors protect motor neurons in an organoid model of Spinal Muscular Atrophy. Cell Death Dis. *9*, 1100. https://doi.org/10.1038/s41419-018-1081-0.

Ikenaka, K., Ishigaki, S., Iguchi, Y., Kawai, K., Fujioka, Y., Yokoi, S., Abdelhamid, R.F., Nagano, S., Mochizuki, H., Katsuno, M., and Sobue, G. (2020). Characteristic features of FUS inclusions in spinal motor neurons of sporadic amyotrophic lateral sclerosis. J. Neuropathol. Exp. Neurol. *79*, 370–377. https://doi.org/10.1093/jnen/nlaa003.

Jaiswal, M.K. (2019). Riluzole and edaravone: a tale of two amyotrophic lateral sclerosis drugs. Med. Res. Rev. *39*, 733–748. https://doi.org/10.1002/med.21528.

Jiang, M., and Chen, G. (2006). High Ca2+-phosphate transfection efficiency in low-density neuronal cultures. Nat. Protoc. *1*, 695–700. https://doi.org/10.1038/nprot.2006.86.

Kawahara, Y., and Mieda-Sato, A. (2012). TDP-43 promotes microRNA biogenesis as a component of the Drosha and Dicer complexes. Proc. Natl. Acad. Sci. USA *109*, 3347–3352. https://doi.org/10.1073/pnas.1112427109.

Kent, W.J., Sugnet, C.W., Furey, T.S., Roskin, K.M., Pringle, T.H., Zahler, A.M., and Haussler, D. (2002). The human genome browser at UCSC. Genome Res. *12*, 996–1006. https://doi.org/10.1101/gr.229102.

Korobeynikov, V.A., Lyashchenko, A.K., Blanco-Redondo, B., Jafar-Nejad, P., and Shneider, N.A. (2022). Antisense oligonucleotide silencing of FUS expression as a therapeutic approach in amyotrophic lateral sclerosis. Nat. Med. *28*, 104–116. https://doi.org/10.1038/s41591-021-01615-z.

Kurita, H., Yabe, S., Ueda, T., Inden, M., Kakita, A., and Hozumi, I. (2020). MicroRNA-5572 is a novel MicroRNA-regulating SLC30A3 in sporadic amyotrophic lateral sclerosis. Int. J. Mol. Sci. *21*, 4482. https://doi.org/10.3390/ijms21124482.

Liberzon, A., Subramanian, A., Pinchback, R., Thorvaldsdóttir, H., Tamayo, P., and Mesirov, J.P. (2011). Molecular signatures database (MSigDB) 3.0. Bioinformatics *27*, 1739–1740. https://doi.org/10.1093/bioinformatics/btr260.

Ling, S.-C., Albuquerque, C.P., Han, J.S., Lagier-Tourenne, C., Tokunaga, S., Zhou, H., and Cleveland, D.W. (2010). ALS-associated mutations in TDP-43 increase its stability and promote TDP-43 complexes with FUS/TLS. Proc. Natl. Acad. Sci. U S A *107*, 13318–13323. https://doi.org/10.1073/pnas.1008227107.

Long, H., Sun, B., Cheng, L., Zhao, S., Zhu, Y., Zhao, R., and Zhu, J. (2017). miR-139-5p represses BMSC osteogenesis via targeting wnt/β-catenin signaling pathway. DNA Cell Biol. *36*, 715–724. https://doi.org/10.1089/dna.2017.3657.

Luisier, R., Tyzack, G.E., Hall, C.E., Mitchell, J.S., Devine, H., Taha, D.M., Malik, B., Meyer, I., Greensmith, L., Newcombe, J., et al. (2018). Intron retention and nuclear loss of SFPQ are molecular hallmarks of ALS. Nat. Commun. *9*, 2010. https://doi.org/10.1038/s41467-018-04373-8.

Morlando, M., Dini Modigliani, S., Torrelli, G., Rosa, A., Di Carlo, V., Caffarelli, E., and Bozzoni, I. (2012). FUS stimulates microRNA biogenesis by facilitating co-transcriptional Drosha recruitment. EMBO J. *31*, 4502–4510. https://doi.org/10.1038/emboj.2012.319.

Purro, S.A., Galli, S., and Salinas, P.C. (2014). Dysfunction of Wnt signaling and synaptic disassembly in neurodegenerative diseases. J. Mol. Cell Biol. *6*, 75–80. https://doi.org/10.1093/jmcb/mjt049.

Qu, B., Han, X., Tang, Y., and Shen, N. (2012). A novel vector-based method for exclusive overexpression of star-form microRNAs. PLoS One *7*, e41504. https://doi.org/10.1371/journal.pone.0041504.

Raheja, R., Regev, K., Healy, B.C., Mazzola, M.A., Beynon, V., Von Glehn, F., Paul, A., Diaz-Cruz, C., Gholipour, T., Glanz, B.I., et al. (2018). Correlating serum micrornas and clinical parameters in amyotrophic lateral sclerosis. Muscle Nerve *58*, 261–269. https://doi.org/10.1002/mus.26106.

Ranganathan, S., and Bowser, R. (2010). p53 and cell cycle proteins participate in spinal motor neuron cell death in ALS~!2010-02-16~!2010-02-19~!2010-04-14~!. Open Pathol. J. *4*, 11–22. https://doi.org/10.2174/1874375701004010011.

Reichenstein, I., Eitan, C., Diaz-Garcia, S., Haim, G., Magen, I., Siany, A., Hoye, M.L., Rivkin, N., Olender, T., Toth, B., et al. (2019). Human genetics and neuropathology suggest a link between miR-218 and amyotrophic lateral sclerosis pathophysiology. Sci. Transl. Med. *11*, eaav5264. https://doi.org/10.1126/scitranslmed.aav5264.

Rojas-Prats, E., Martinez-Gonzalez, L., Gonzalo-Consuegra, C., Liachko, N.F., Perez, C., Ramírez, D., Kraemer, B.C., Martin-Requero, Á., Perez, D.I., Gil, C., et al. (2021). Targeting nuclear protein TDP-43 by cell division cycle kinase 7 inhibitors: a new therapeutic approach for amyotrophic lateral sclerosis. Eur. J. Med. Chem. *210*, 112968. https://doi.org/10.1016/j.ejmech.2020.112968.

Ryu, Y.K., Collins, S.E., Ho, H.-Y.H., Zhao, H., and Kuruvilla, R. (2013). An autocrine Wnt5a-Ror signaling loop mediates sympathetic target innervation. Dev. Biol. *377*, 79–89. https://doi.org/10.1016/j.ydbio.2013.02.013.

Sama, R.R.K., Fallini, C., Gatto, R., McKeon, J.E., Song, Y., Rotunno, M.S., Penaranda, S., Abdurakhmanov, I., Landers, J.E., Morfini, G., et al. (2017). ALS-linked FUS exerts a gain of toxic function involving aberrant p38 MAPK activation. Sci. Rep. *7*, 115. https://doi.org/10.1038/s41598-017-00091-1.

Schlange, T., Matsuda, Y., Lienhard, S., Huber, A., and Hynes, N.E. (2007). Autocrine WNT signaling contributes to breast cancer cell proliferation via the canonical WNT pathway and EGFR transactivation. Breast Cancer Res. *9*, R63. https://doi.org/10.1186/bcr1769.

Schwartz, J.C., Ebmeier, C.C., Podell, E.R., Heimiller, J., Taatjes, D.J., and Cech, T.R. (2012). FUS binds the CTD of RNA polymerase II and regulates its phosphorylation at Ser2. Genes Dev. *26*, 2690–2695. https://doi.org/10.1101/gad.204602.112.

Sellers, K.J., Elliott, C., Jackson, J., Ghosh, A., Ribe, E., Rojo, A.I., Jarosz-Griffiths, H.H., Watson, I.A., Xia, W., Semenov, M., et al. (2018). Amyloid β synaptotoxicity is Wnt-PCP dependent and blocked by fasudil. Alzheimers Dement. *14*, 306–317. https://doi.org/10.1016/j.jalz.2017.09.008.

Sharma, A., Lyashchenko, A.K., Lu, L., Nasrabady, S.E., Elmaleh, M., Mendelsohn, M., Nemes, A., Tapia, J.C., Mentis, G.Z., and Shneider, N.A. (2016). ALS-associated mutant FUS induces selective motor neuron degeneration through toxic gain of function. Nat. Commun. *7*, 10465. https://doi.org/10.1038/ncomms10465.

Sticht, C., De La Torre, C., Parveen, A., and Gretz, N. (2018). miRWalk: an online resource for prediction of microRNA binding sites. PLoS One *13*, e0206239. https://doi.org/10.1371/journal.pone.0206239.

Subramanian, A., Tamayo, P., Mootha, V.K., Mukherjee, S., Ebert, B.L., Gillette, M.A., Paulovich, A., Pomeroy, S.L., Golub, T.R., Lander, E.S., and Mesirov, J.P. (2005). Gene set enrichment analysis: a knowledge-based approach for interpreting genome-wide expression profiles. Proc. Natl. Acad. Sci. U S A *102*, 15545–15550. https://doi.org/10.1073/pnas.0506580102.

Tank, E.M., Figueroa-Romero, C., Hinder, L.M., Bedi, K., Archbold, H.C., Li, X., Weskamp, K., Safren, N., Paez-Colasante, X., Pacut, C., et al. (2018). Abnormal RNA stability in amyotrophic lateral sclerosis. Nat. Commun. *9*, 2845. https://doi.org/10.1038/s41467-018-05049-z.

Tung, Y.-T., Peng, K.-C., Chen, Y.-C., Yen, Y.-P., Chang, M., Thams, S., and Chen, J.-A. (2019). Mir-17~92 confers motor neuron subtype differential resistance to ALS-associated degeneration. Cell Stem Cell *25*, 193–209.e7. https://doi.org/10.1016/j.stem.2019.04.016.

Tu, Y., Gao, X., Li, G., Fu, H., Cui, D., Liu, H., Jin, W., and Zhang, Y. (2013). MicroRNA-218 inhibits glioma invasion, migration, proliferation, and cancer stem-like cell self-renewal by targeting the polycomb group gene Bmi1. Cancer Res. *73*, 6046–6055. https://doi.org/10.1158/0008-5472.can-13-0358.

Wang, Y., Huang, C., Reddy Chintagari, N., Bhaskaran, M., Weng, T., Guo, Y., Xiao, X., and Liu, L. (2013). miR-375 regulates rat alveolar epithelial cell trans-differentiation by inhibiting Wnt/β-catenin pathway. Nucleic Acids Res. *41*, 3833–3844. https://doi.org/10.1093/nar/gks1460.

Wexler, E.M., Paucer, A., Kornblum, H.I., Palmer, T.D., and Geschwind, D.H. (2009). Endogenous Wnt signaling maintains neural progenitor cell potency. Stem Cell. *27*, 1130–1141. https://doi.org/10.1002/stem.36.

Wishart, T.M., Mutsaers, C.A., Riessland, M., Reimer, M.M., Hunter, G., Hannam, M.L., Eaton, S.L., Fuller, H.R., Roche, S.L., Somers,

E., et al. (2014). Dysregulation of ubiquitin homeostasis and β-catenin signaling promote spinal muscular atrophy. J. Clin. Invest. *124*, 1821–1834. https://doi.org/10.1172/jci71318.

Wong, N., and Wang, X. (2015). miRDB: an online resource for microRNA target prediction and functional annotations. Nucleic Acids Res. *43*, D146–D152. https://doi.org/10.1093/nar/gku1104.

Xu, L., Liu, T., Liu, L., Yao, X., Chen, L., Fan, D., Zhan, S., and Wang, S. (2020). Global variation in prevalence and incidence of amyotrophic lateral sclerosis: a systematic review and meta-analysis. J. Neurol. *267*, 944–953. https://doi.org/10.1007/s00415-019-09652-y.

Yu, L., Guan, Y., Wu, X., Chen, Y., Liu, Z., Du, H., and Wang, X. (2013). Wnt Signaling is altered by spinal cord neuronal dysfunction in amyotrophic lateral sclerosis transgenic mice. Neurochem. Res. *38*, 1904–1913. https://doi.org/10.1007/s11064-013-1096-y.

Zhang, T., Wu, Y.-C., Mullane, P., Ji, Y.J., Liu, H., He, L., Arora, A., Hwang, H.-Y., Alessi, A.F., Niaki, A.G., et al. (2018). FUS regulates activity of MicroRNA-mediated gene silencing. Mol. Cell *69*, 787–801.e8. https://doi.org/10.1016/j.molcel.2018.02.001.

Zhao, B., Chen, Y., Yang, N., Chen, Q., Bao, Z., Liu, M., Hu, S., Li, J., and Wu, X. (2019). miR-218-5p regulates skin and hair follicle development through Wnt/β-catenin signaling pathway by targeting SFRP2. J. Cell. Physiol. *234*, 20329–20341. https://doi.org/10.1002/jcp.28633.

Zou, F., Mao, R., Yang, L., Lin, S., Lei, K., Zheng, Y., Ding, Y., Zhang, P., Cai, G., Liang, X., and Liu, J. (2016). Targeted deletion of miR-139-5p activates MAPK, NF-κB and STAT3 signaling and promotes intestinal inflammation and colorectal cancer. FEBS J. *283*, 1438–1452. https://doi.org/10.1111/febs.13678.

Stem Cell Reports, Volume *17*

Supplemental Information

Upregulation of β-catenin due to loss of miR-139 contributes to motor neuron death in amyotrophic lateral sclerosis

Sophie Hawkins, Seema C. Namboori, Ammarah Tariq, Catherine Blaker, Christine Flaxman, Nidhi S. Dey, Peter Henley, Andrew Randall, Alessandro Rosa, Lawrence W. Stanton, and Akshay Bhinge

Figure S1

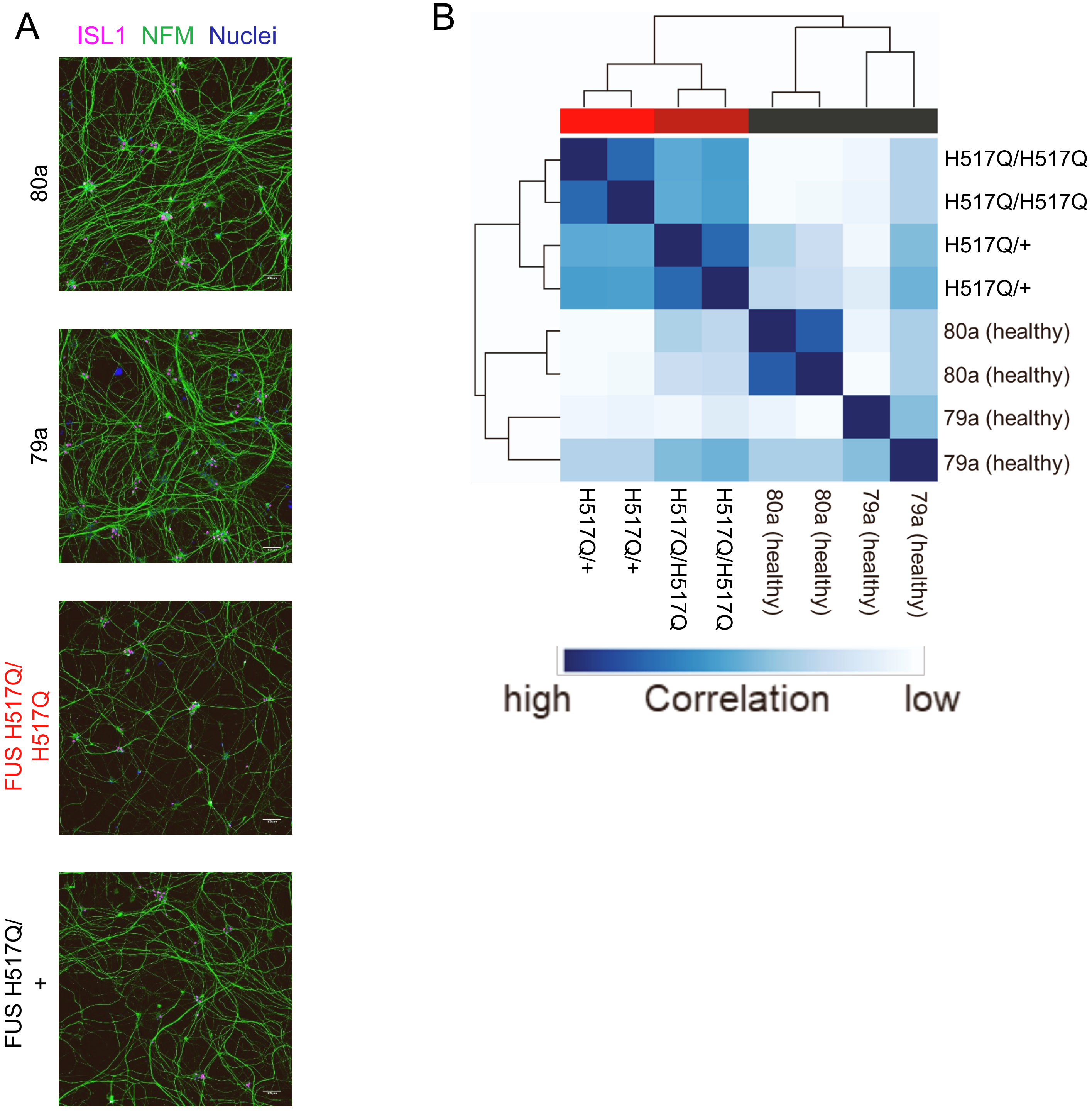

Figure S1. Related to figures 1 and 2.
A) Representative images of healthy (80a and 79a) MNs and FUS ALS (FUS H517Q/H517Q and FUS H517Q/+) at day 45. Scale bar indicates 100um. **B).** Hierarchical cluster analysis of 844 miRNAs in FUS H517Q/H517Q, FUS H517Q/+ and healthy MNs. N = 2 independent differentiations.

Figure S2

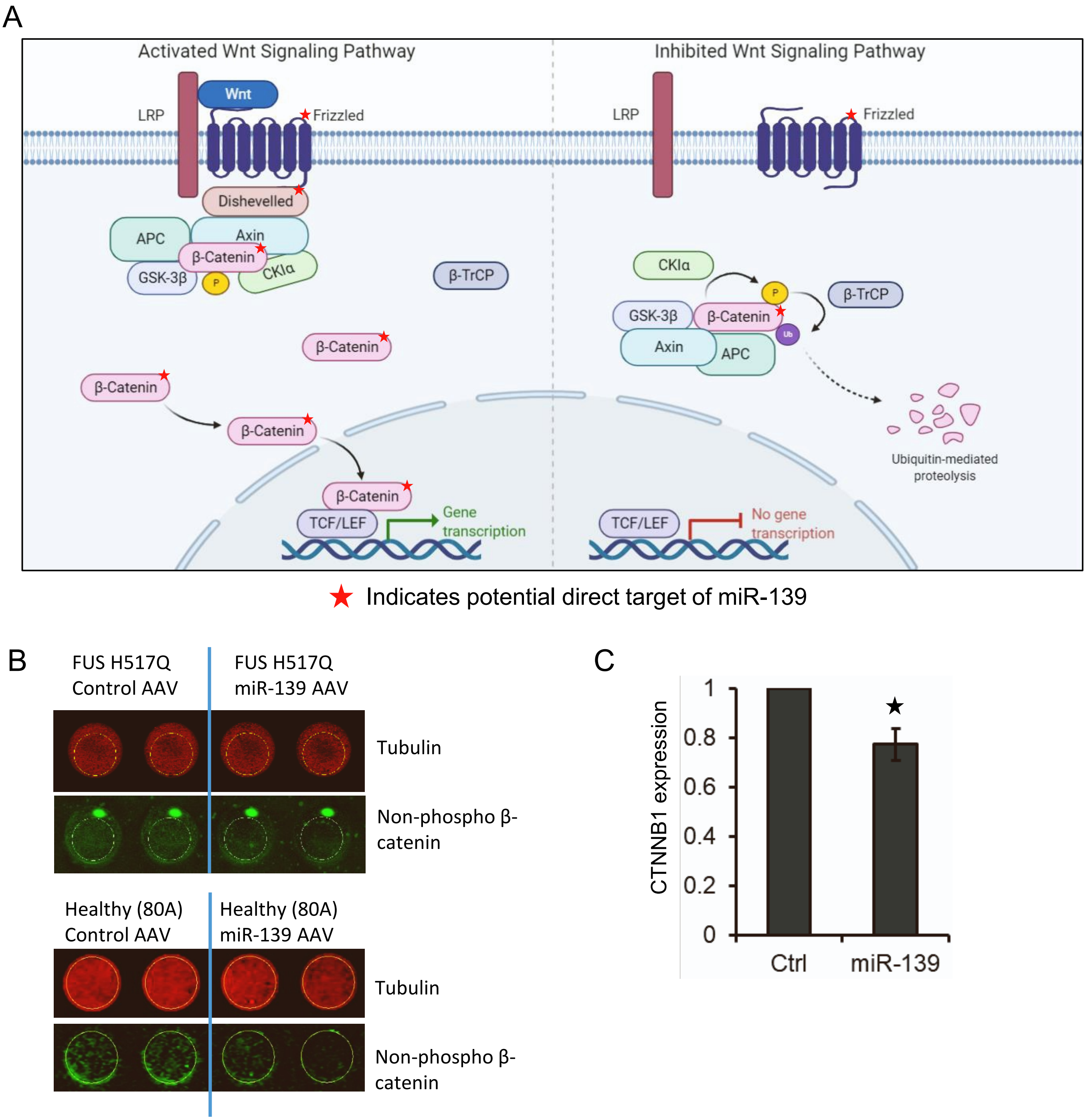

Figure S2. Related to figure 3.
A) Schematic of activated and inhibited canonical WNT signalling (left and right panels respectively). Canonical WNT signalling is activated by the binding of WNT ligands to frizzled and LRP5/6 co-receptors on the cell surface. This leads to the recruitment and sequestration of the β-catenin destruction complex to the membrane, preventing further phosphorylation and ubiquitin-mediated destruction. β-catenin accumulates and translocates to the nucleus where it binds to TCF and promotes transcription of downstream target genes. Image created with Biorender. Stars indicate the top three potential direct targets of miR-139 displayed in Fig.3E. **B)** Representative images of in-cell western blots in figure 3F. The circles indicate the area used for quantification. **C)** RT-qPCR analysis of CTNNB1 / β-catenin transcript in Day 34 FUS H517Q/H517Q MNs in response to miR-139 overexpression. N = 3 independent differentiations. Error bars indicate SEM. * indicates p-value < 0.05.

Figure S3

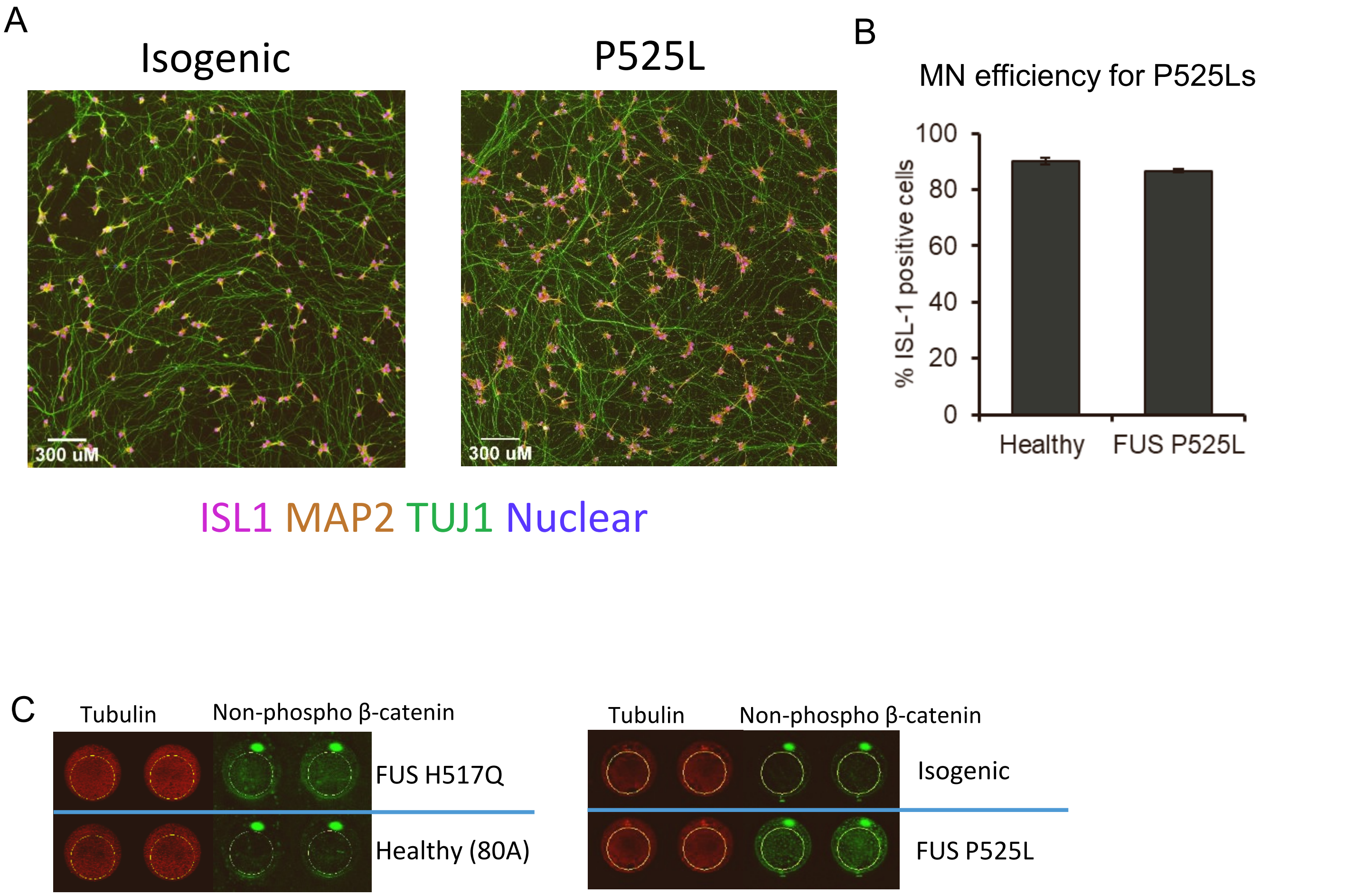

Figure S3: Related to figure 4

A) Representative images: Isogenic and P525L MNs immunostained with ISL1, MAP2 and TUJ1. Scale bar indicates 300uM. **B)** MN differentiation efficiency, determined by ISL-1+ nuclei. N = 3 independent differentiations. **C)** Representative images for in-cell western blot. Left panel = FUS H517Q/H517Q vs healthy MNs (80A). Right panel = FUS P525L vs isogenic control MNs. Circles indicate area used for quantification.

Figure S4

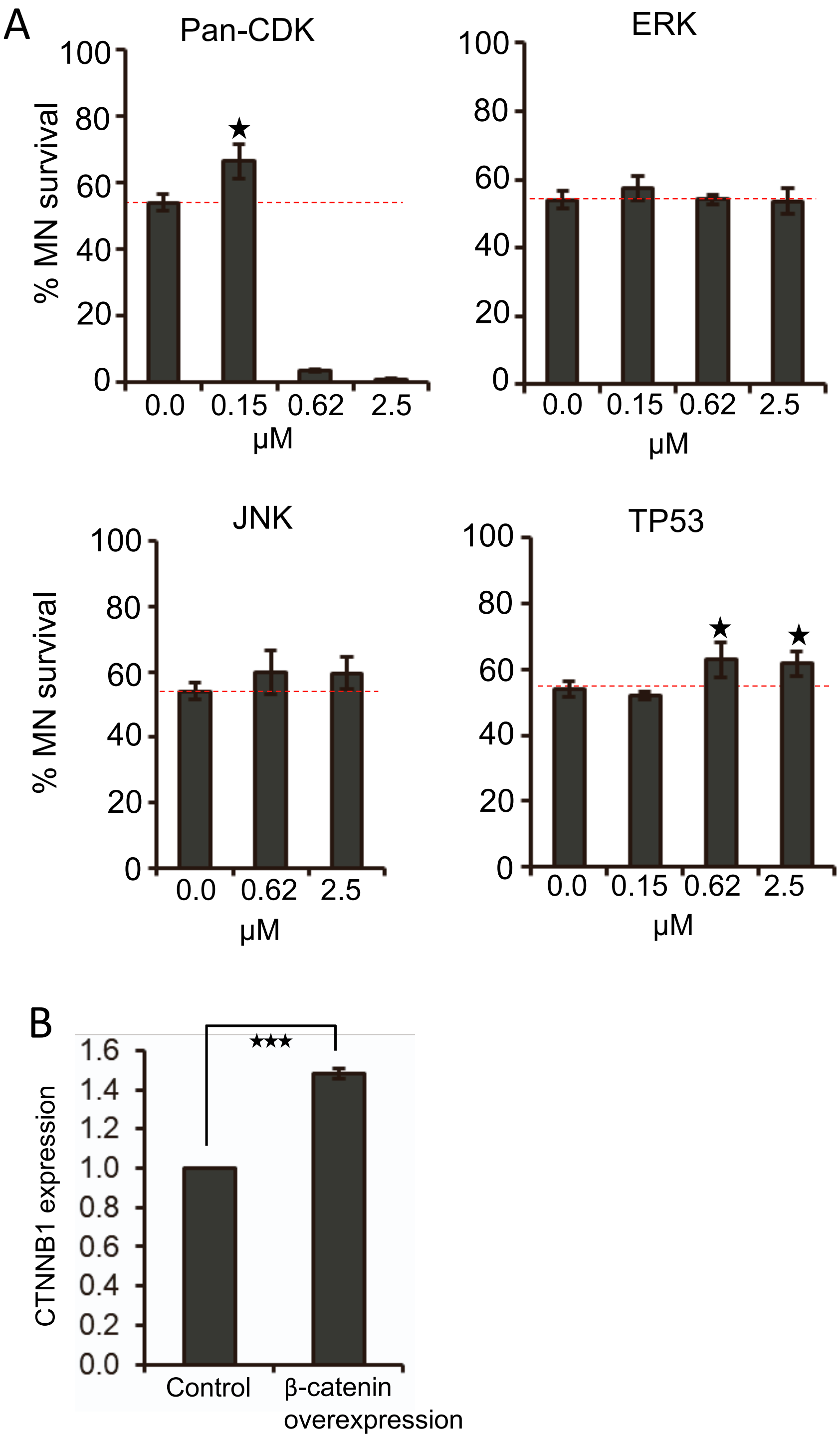

Figure S4: Related to figure 4, role of WNT in FUS ALS MN degeneration
A) Quantification of ISL1 positive FUS H517Q/H517Q MN at day 45 relative to day 30 after treatment with small molecule inhibitors of the indicated pathways. Horizontal dotted line indicates MN loss in the DMSO only control. N = 3 independent differentiations. **B)** Quantitative RT-qPCR indicating upregulation of CTNNB1 transcript 48 hours post-transduction in healthy (80A) MN progenitors. N = 3.
N indicates the number of independent differentiations. * indicates p-value < 0.05, ** indicates p-value < 0.01. P-values were estimated by Student's two-tailed t-test.

Figure S5

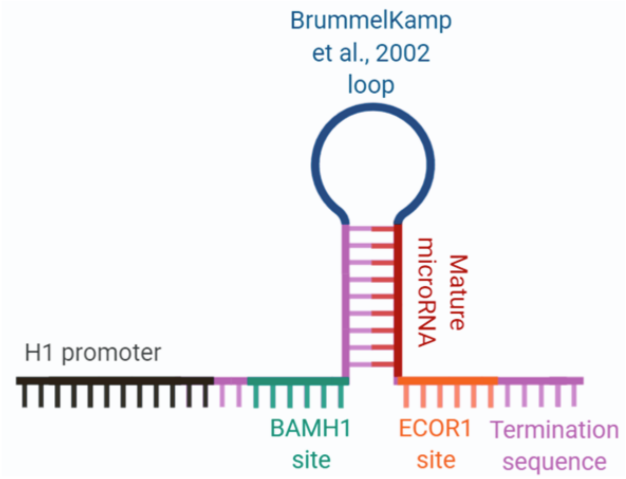

Figure S5. Schematic depicting shRNA design for miR-139-5p overexpression

Antibody	Species	Brand / identifier	Dilution
ISL-1	Ms / Rb	Abcam ab86501 / ab108517	1:500
NF-H	Rb	Sigma N4142	1:1000
MAP2	Ms / Rb	Abcam ab11267 / ab32454	1:1000
TUJ1	Ck	Abcam ab107216	1:1000
Total β-catenin	Rb	Abcam ab16051	1:500<br>1:4000
Non-phospo β-catenin	Rb	CST 8814	1:500
Tubulin	Ms	Abcam ab7291	1:1000

Table S1. Primary antibodies used for immunofluorescence and western blot

Target	Forward 5' to 3'	Reverse 5' to 3'
AXIN2	GTGAGGTCCACGGAAACTGT	TGGCTGGTGCAAAGACATAG
DKK1	CCTTGGATGGGTATTCCAGA	CCTGAGGCACAGTCTGATGA
CCND3	TTGCACATGATTTCCTGGCC	ACTTCAGTGCCAGTGATCCC
WNT3	CGCACGACTATCCTGGAC	GAGGCGCTGTCATACTTGTC
WNT4	CTAGCCCCGACTTCTGTGAG	TTGGACGTCTTGTTGCATGT
WNT5B	CGGGAGCGAGAGAAGAACT	CGTCTGCCATCTTATACACAGC
WNT10B	GATACCCACAACCGCAATTC	GGGTCTCGCTCACAGAAGTC
WNT11	GGCCAAGTTTTCCGATGCTC	CACCCCATGGCACTTACACT
CTNNB1	CACAAGCAGAGTGCTGAAGGTG	GATTCCTGAGAGTCCAAAGACAG
HPRT1	CATTATGCTGAGGATTTGGAAAGG	CTTGAGCACACAGAGGGCTACA
RPL13	CCTGGAGGAGAAGAGGAAAGAGA	TTGAGGACCTCTGTGTATTTGTCAA

Table S2. Primer sequences for mRNA qPCR

Target	5' to 3'
Universal R	GCATAGACCTGAATGGCGGTA
hsa-miR-139-5p	CAGTGCACGTGTCTCCAGTAAAA
SNORD44	GCAAATGCTGACTGAACATGAA
SNORD48	CTCTGAGTGTGTCGCTGATGC
RNU19	GGAATGACTCCTGTGGAGTTGA

Table S3. Primer sequences for microRNA qPCR

Chemical	Source	Pathway / Target
Pifithrin-α hydrobromide	Tocris	TP53
SB203580	Tocris	p38/MAPK
FR180204	SCBT	ERK 1,2
SP600125	SCBT	JNK 1,2,3
XAV 939	Tocris	WNT
AT7519	Selleckchem	CDK1,2,3,4,6,9

Table S4. Small molecule inhibitors

Supplemental experimental procedures

Human iPSC culture

ALS patient-derived FUS H517Q/H517Q iPSC (ND35663), and healthy iPSCs (GM23280A, GM23279A) were obtained from the Coriell Institute for Medical Research. FUS H517Q/H517Q iPSCs were genome edited using CRISPR to generate the FUS H517Q/+ heterozygous cell line. All iPSC lines were maintained as colonies on human ES qualified Matrigel (Corning) in StemFlex (StemCell Technologies). Colonies were routinely passaged in a 1:6 split using EDTA. Mycoplasma testing was conducted regularly to rule out contamination of cultures.

Differentiation of iPSC into spinal motor neurons

iPSC were plated as colonies onto Matrigel and differentiated by treatment with neuronal differentiation media (DMEM/F12:Neurobasal in a 1:1 ratio, HEPES 10mM, N2 supplement 1%, B27 supplement 1%, L-glutamine 1%, ascorbic acid 5uM, insulin 20ug/ml) supplemented with SB431542 (40uM), CHIR9921 (3uM) and LDN8312 (0.2uM) from day 0 till day 4. Cells were caudalized by treatment with 0.1uM retinoic acid starting at day 2 and ventralized with 1uM purmorphamine starting at day 4 and continued till day 8. At day 8, motor neuron progenitors were re-plated onto poly-D-lysine/laminin coated wells and differentiation was induced by treating the cells with N2B27 media supplemented with retinoic acid, purmorphamine and and DAPT 10uM. DAPT treatment was stopped at day 13 and media was changed to N2B27 supplemented with BDNF 10ng/ml, GDNF 10ng/ml. Next day (day 14) neuronal cultures were pulsed with mitomycin at a dose of 10ug/ml for 1 hour to prevent further proliferation of any undifferentiated progenitors. Neuronal cultures were maintained by changing half the media every 2-3 days.

Intracellular Ca^{2+} imaging

Day 30 MNs were plated onto poly-D-lysine and laminin coated cover slips that had been sterilized by autoclaving. Cells were loaded with the Ca^{2+} sensitive dye Fura2-AM at a final concentration of 4μg/mL for 60 min at 37°C in a HEPES-buffered saline solution (HBSS) consisting of (140mM NaCl, 3mM KCl, 2mM CaCl2, 1mM MgCl2, 2.5mM Glucose, 10mM HEPES-NaOH, pH 7.35).

Coverslips were placed in a chamber on the stage of an inverted microscope (Nikon, 11 Eclipse TE2000-S). The cells were imaged (20x magnification) whilst constantly being perfused with HBSS (~1.4 mL/min). Every 5 seconds, Fura-2 was excited in succession at wavelengths of 340 and 380 nm using a high speed wavelength switcher (Lambda DG-4); the emitted fluorescence images at 510 nM were collected using a CCD camera (Hamamatsu Photonics). After a baseline period, neurons were treated for 2 min with 50μM glutamate dissolved the perfusing HBSS solution. The timeseries of the emission ratio from 340 and 380 excitation, which is proportion to intracellular Ca^{2+}, was calculated as described previously (Telezhkin *et al*, 2016). Data was collected in Volocity software (PerkinElmer) and plotted in Origin Pro.

Immunostaining

Cells were fixed with 4% paraformaldehyde, permeabilized with ice-cold methanol for 5 minutes and washed with PBS containing 10% serum for 1 hour at room temperature. Cells were incubated with primary antibodies (Table S1) diluted into PBS containing 1% BSA and incubated overnight at 4°C. Next day, cells were washed and incubated with Alexa-fluor conjugated secondary antibodies (Molecular probes) for 45 minutes at room temperature and nuclei were stained with Hoechst 33542 (Molecular probes). Images were obtained in an automated fashion on the ImageXpress Pico (Molecular devices). Nuclear identification and ISL1 nuclear signal was quantified in an automated fashion using ImageXpress software. Threshold intensities were maintained the same across wells.

MN survival assay

Motor neuron progenitors were plated in 96-well optically clear black tissue culture plates (Greiner). Day 30 cultures were fixed and stained for ISL1 to assess MN counts. A separate plate cultured under the same condition was allowed to proceed till day 45, when it was fixed and stained for ISL1. Nuclei were stained using Hoechst 33342. MN counts at day 45 were compared with day 30 to assess MN survival. We performed three independent differentiations with two technical wells used per replicate. Data from the technical wells was pooled to generate counts for each replicate. Apoptotic cells at day 37 were detected using the CellEvent Caspase 3/7 green detection reagent (Thermofisher) according to manufacturer's instructions. Drug treatments (Table S4) were initiated at day 30 and MN survival performed as described above.

Quantitative RT-PCR

Total RNA was extracted with the miRNeasy kit (Qiagen) or Monarch Total RNA miniprep (NEB) and reverse transcribed using random hexamers and the High Capacity reverse transcription system from Applied Biosystems. Quantitative PCR was performed using the SYBR GREEN PCR Master Mix from Applied Biosystems. The target gene mRNA expression was normalized to the expression of two housekeeping genes (HPRT1 and RPL13), and relative mRNA fold changes were calculated by the ΔΔCt method. Primer sequences are included in Table S2.

Western blot analysis

Cell lysates were prepared in RIPA buffer, separated on 12% SDS-PAGE gels and proteins were transferred onto PVDF membranes. Membranes were blocked with 5% milk in TBST (25 mM Tris pH 8.0, 150 mM NaCl, 0.05% Tween-20) and probed with corresponding primary antibodies against specific proteins (Table S1). Dylight 680 and 800 secondary antibodies

(Cell signalling technology) were used to detect primary antibodies and proteins were visualized by using fluorescence (Licor).

In-cell western assay

Cells were fixed with 4% paraformaldehyde, permeabilized with ice-cold methanol for 5 minutes and washed with PBS containing 10% serum for 1 hour at room temperature. Cells were incubated with primary antibodies (Table S1) diluted into PBS containing 1% BSA and incubated overnight at 4°C. Next day, cells were washed and incubated with Dylight 680 and 800 secondary antibodies (Cell signalling technology) for 1 hr at room temperature. Fluorescence was captured with the Odyssey CLx Imaging System (LI-COR) according to the manufacturer's instructions.

MicroRNA RT-qPCR

Total RNA was reverse transcribed using the qScript microRNA cDNA Synthesis Kit (QuantaBio). Quantitative PCR was performed with SYBR GREEN PCR Master Mix from Applied Biosystems and fold changes were calculated by the ΔΔCt method. Primer sequences are included in Table S3.

Caspase 3/7 Assay

Caspase was measured using the Promega Caspase-Glo® 3/7 Assay System kit (G8090). Caspase luminescence is presented relative to cell titer, as measured by Promega CellTiter-Glo® 2.0 Cell Viability Assay.

FUS Knockdown

300,000 SH-SY5Y or MN progenitor cells were nucleofected with 30 pmol silencer select siRNA (Thermo Fisher) against FUS or appropriate scrambled control using the P3 Primary nucleofector kit (Lonza).

RNA sequencing and analysis

RNA was extracted from day 30 neuronal cultures as described above. MicroRNA sequencing libraries were generated from 500ng of RNA using the NEBnext Small RNA kit (NEB) according to manufacturer's instructions and sequenced on an Illumina MiSeq instrument. Raw reads were trimmed to remove adapter sequences, and PCR duplicates collapsed using the fastx toolkit (http://hannonlab.cshl.edu/fastx_toolkit/index.html). Processed reads were mapped to the microRNA hairpin sequences obtained from miRbase using bowtie (Langmead *et al*, 2009) and custom scripts were used to generate counts per microRNA.

Total RNA extracted as described above was used to generate Illumina libraries for whole transcriptome analysis using the NEBNext Ultra II kit (NEB). RNA-sequencing reads were trimmed using cutadapt (Martin, 2011) and mapped to the human genome hg19 using bwa (Li & Durbin, 2009). Only reads mapping to unique locations were retained for further analysis.

For the miRNA OE analysis, total RNA was extracted at day 35. RNA was processed using the Quant-seq kit (Lexogen) to generate 3'end libraries compatible with Illumina. Reads were trimmed using cutadapt and mapped to the hg19 using STAR (Dobin *et al*, 2013).

The DESeq2 R package (Love *et al*, 2014) and custom R scripts were used to analyze the read counts and generate a list of differentially expressed genes sorted by the statistical score. Gene set enrichment analysis was performed using the GSEA software (http://software.broadinstitute.org/gsea/index).

Pri-miRNA quantification

We sought to determine pri-miRNA transcript levels from RNA-seq data. Over 60% of all mapped miRNAs to date are intragenic, residing within introns of host genes (Hinske *et al*, 2017). Since most intragenic miRNAs display correlated expression with their host genes (Liu

et al, 2018), we decided to use the host gene expression as a proxy for pri-miRNA expression levels. Standard RNA-seq experiments generate reads that can be classified as intronic or exonic based on whether they map to introns or exons respectively. Since introns are absent in cytosolic mRNAs, RNA-Seq reads mapping to introns can be used to represent levels of unspliced transcripts present in the nucleus. Changes in intronic read counts can be used to measure changes in transcriptional output for a given gene across experimental conditions (Gaidatzis *et al*, 2015). Hence, we measured intronic reads mapping to host genes as estimates of pri-miRNA transcription.

Intronic miRNAs were identified from the miRIAD intragenic microRNA database (Hinske *et al*, 2014). MiRNAs and host genes were shortlisted to those that were expressed in our MN datasets. Custom R scripts were used to quantify reads mapping to intronic regions of host genes from whole transcriptome libraries. Reads were normalised using scale factors generated with DESeq2.

References

Dobin A, Davis CA, Schlesinger F, Drenkow J, Zaleski C, Jha S, Batut P, Chaisson M & Gingeras TR (2013) STAR: ultrafast universal RNA-seq aligner. *Bioinformatics* 29: 15–21

Gaidatzis D, Burger L, Florescu M & Stadler MB (2015) Analysis of intronic and exonic reads in RNA-seq data characterizes transcriptional and post-transcriptional regulation. *Nat Biotechnol* 33: 722–729

Hinske LC, Dos Santos FRC, Ohara DT, Ohno-Machado L, Kreth S & Galante PAF (2017) MiRIAD update: using alternative polyadenylation, protein interaction network analysis and additional species to enhance exploration of the role of intragenic miRNAs and their host genes. *Database* 2017

Hinske LC, França GS, Torres HAM, Ohara DT, Lopes-Ramos CM, Heyn J, Reis LFL, Ohno-Machado L, Kreth S & Galante PAF (2014) miRIAD-integrating microRNA inter- and intragenic data. *Database* 2014

Langmead B, Trapnell C, Pop M & Salzberg SL (2009) Ultrafast and memory-efficient alignment of short DNA sequences to the human genome. *Genome Biol* 10: R25

Li H & Durbin R (2009) Fast and accurate short read alignment with Burrows-Wheeler transform. *Bioinformatics* 25: 1754–1760

Liu B, Shyr Y, Cai J & Liu Q (2018) Interplay between miRNAs and host genes and their role

in cancer. *Brief Funct Genomics* 18: 255–266

Love MI, Huber W & Anders S (2014) Moderated estimation of fold change and dispersion for RNA-seq data with DESeq2. *Genome Biol* 15: 550

Martin M (2011) Cutadapt removes adapter sequences from high-throughput sequencing reads. *EMBnet.journal* 17: 10–12

Telezhkin V, Schnell C, Yarova P, Yung S, Cope E, Hughes A, Thompson BA, Sanders P, Geater C, Hancock JM, *et al* (2016) Forced cell cycle exit and modulation of GABAA, CREB, and GSK3β signaling promote functional maturation of induced pluripotent stem cell-derived neurons. *Am J Physiol Cell Physiol* 310: C520–41
